# Association of a Chromosomal Rearrangement Event with Mouse Posterior Polymorphous Corneal Dystrophy and Alterations in *Csrp2bp*, *Dzank1*, and *Ovol2* Gene Expression

**DOI:** 10.1371/journal.pone.0157577

**Published:** 2016-06-16

**Authors:** Anna L. Shen, Susan A. Moran, Edward A. Glover, Norman R. Drinkwater, Rebecca E. Swearingen, Leandro B. Teixeira, Christopher A. Bradfield

**Affiliations:** 1 The McArdle Laboratory for Cancer Research, Department of Oncology, School of Medicine and Public Health, University of Wisconsin-Madison, Madison, WI, United States of America; 2 Department of Pathobiological Sciences, School of Veterinary Medicine, University of Wisconsin-Madison, Madison, WI, United States of America; 3 Wisconsin Institute for Discovery, University of Wisconsin-Madison, Madison, WI, United States of America; 4 McPherson Eye Research Institute, University of Wisconsin, Madison, Wisconsin, United States of America; University of Iowa, UNITED STATES

## Abstract

We have previously described a mouse model of human posterior polymorphous corneal dystrophy (PPCD) and localized the causative mutation to a 6.2 Mbp region of chromosome 2, termed *Ppcd1*. We now show that the gene rearrangement linked to mouse *Ppcd1* is a 3.9 Mbp chromosomal inversion flanked by 81 Kbp and 542 bp deletions. This recombination event leads to deletion of *Csrp2bp* Exons 8 through 11, *Dzank1* Exons 20 and 21, and the pseudogene *Znf133*. In addition, we identified translocation of novel downstream sequences to positions adjacent to *Csrp2bp* Exon 7 and *Dzank1* Exon 20. Twelve novel fusion transcripts involving *Csrp2bp* or *Dzank1* linked to downstream sequences have been identified. Eight are expressed at detectable levels in PPCD1 but not wildtype eyes. Upregulation of two *Csrp2bp* fusion transcripts, as well as upregulation of the adjacent gene, *Ovol2*, was observed. Absence of the PPCD1 phenotype in animals haploinsufficient for *Csrp2bp* or both *Csrp2bp* and *Dzank1* rules out haploinsufficiency of these genes as a cause of mouse PPCD1. Complementation experiments confirm that PPCD1 embryonic lethality is due to disruption of *Csrp2bp* expression. The ocular expression pattern of *Csrp2bp* is consistent with a role for this protein in corneal development and pathogenesis of PPCD1.

## Introduction

Posterior polymorphous corneal dystrophy (PPCD, OMIM #122000) is a rare corneal endothelial dystrophy characterized by the presence of multilayered, metaplastic corneal endothelial cells which display proliferative capacity and epithelial features including microvilli and inappropriate cytokeratin expression [1–4]. Clinical outcomes vary from minimal visual impairment to development of corneal edema, retrocorneal membranes, corneal opacification requiring keratoplasty, and increased intraocular pressure and development of glaucoma as a result of blockage of the iridocorneal angle by proliferating corneal endothelial cells [5, 6]. Recently, PPCD has also been associated with development of astigmatism and steep corneal curvature [7, 8]. The sporadic condition, iridocorneal endothelial syndrome (ICE), bears phenotypic similarity to PPCD and is also associated with increased intraocular pressure and development of glaucoma [9].

Human PPCD has been linked to three chromosomal loci, 20p11.2 (*PPCD1*), 1p34.3-p32 (*PPCD2*, *COL8A2*), and 10p11 (*PPCD3*, *ZEB1*). The human *PPCD3* locus is the best characterized, with truncating mutations in *ZEB1* linked to 9–45% of all PPCD cases depending on the population studied [10–13]. Although Fuch’s corneal endothelial dystrophy (FECD) has not been formally linked to the PPCD3 locus, missense mutations in *ZEB1* have been associated with this condition. COL8A2 has been linked to early-onset FECD [14, 15], but linkage of this gene to PPCD has been disputed [14]. Finally, Liskova *et al*. have proposed that, in the Czech Republic, which has an unusually high prevalence of PPCD, the PPCD1 locus on Chromosome 20 may account for the majority of PPCD cases [16]. Sequencing of coding regions of genes in the human PPCD1 interval as well as next-generation sequencing of the region between D20S48 and D20S190 from a single affected individual had not identified causative mutations [16–18]. However, a recent report has linked mutations in the promoter region of the gene *OVOL2* to human PPCD1[19].

Our laboratory has previously described the PPCD1 mouse, which reproduces a hallmark feature of human PPCD, corneal endothelial metaplasia characterized by proliferation of corneal endothelial cells with epithelial features including ectopic pan-cytokeratin expression (cytokeratin AE1/AE3) [20]. Affected animals exhibit a visibly enlarged anterior chamber. The mouse *Ppcd1* mutation has been localized to a region of mouse chromosome 2 that is syntenic to the human *PPCD1* locus on chromosome 20. Other mouse models that reproduce one or more features of PPCD have also been described. Inactivation of both *Col8A1* and *Col8A2* produces a keratoglobus-like deep anterior chamber with a thin corneal stroma and decreased numbers of corneal endothelial cells.[21, 22]. Mice haploinsufficient for *Zeb1* have been reported to exhibit corneal thickening, increased keratocyte number, and iridocorneal and corneolenticular adhesions [23]. Proliferation and epithelialization of corneal endothelial cells were not observed in either of these models.

We have previously demonstrated that expression of two genes, *Csrp2bp* and *Dzank1*, is disrupted in eyes of PPCD1 mice [20]. Now, we more thoroughly describe the chromosomal rearrangement of the mouse *Ppcd1* locus, characterize expression of candidate genes in this region, and exclude the hypothesis that haploinsufficiency of *Csrp2bp* or *Csrp2bp* and *Dzank1* is the cause of the PPCD1 eye abnormalities.

## Materials and Methods

### Animals

All procedures conformed to the principles embodied in the Association for Research in Vision and Ophthalmology Statement for the Use of Animals in Ophthalmic and Vision Research (www.arvo.org). Procedures were also approved by the Animal Care and Use Committee, School of Medicine and Public Health, University of Wisconsin-Madison (Protocol Number M00578). Animals were housed in groups of two to four in plastic cages with corncob bedding, maintained on a 12-hour light:dark cycle, and provided with lab chow (Mouse diet 9F 5020; PMI Nutrition International) and water ad lib. DBA/2J (D2) and C57BL6/J (B6) mice were obtained from the Jackson Laboratory, Bar Harbor ME. B6N.129S4-*Gt(ROSA)26Sor*^*tm1(FLP1)Dym*^/J (ROSA-flp, JAX 016226) and B6.C-Tg(CMV-cre)1Cgn/J, JAX 006054) were also obtained from the Jackson Laboratory. The PPCD1 mouse has been described previously [20] and is maintained on the D2 background. The *Csrp2bp*^*tm1a(KOMP)Wtsi*^ mouse was obtained from the Wellcome Trust Sanger Institute (International Knockout Mouse Consortium). The *Dzank1* ^*tm1a(KOMP)Mbp*^ mouse was obtained from the KOMP Repository (www.komp.org). The vector, ES cells, and mouse strain for this allele were generated by the trans-NIH Knock-Out Mouse Project (KOMP).

### Statistical Analysis

Statistical analysis was carried out using the software programs Mstat (Dr. Norman Drinkwater, University of Wisconsin-Madison, WI, http://www.mcardle.wisc.edu/mstat/) and GraphPad Prism (GraphPad Software Inc., La Jolla CA). P values were determined using the Kruskal-Wallis test (copy number, qPCR) or chi-squared test (complementation).

### Isolation and sequencing of BAC clones

A BAC library was prepared and screened by the Clemson University Genomics Institute using PPCD1 liver DNA, as described [24]. Briefly, nuclei were isolated and resuspended in 0.75% Seaplaque low melting point agarose in phosphate buffered saline and dispensed into nuclei plug molds (Bio-Rad, Hercules CA). High molecular weight DNA isolation, Hind III partial digestion, DNA size selection, and preparation of the pIndigoBAC536 cloning vector were conducted according to the methods of Luo and Wing [25]. The size selected high molecular fragments were ligated to the vector and transformed into *E*. *coli* strain DH10B (Invitrogen, Carlsbad CA). Recombinant colonies (5,000 per pool) were selected on LB agar plates (22cm X 22cm) supplemented with 12.5 ug/mL chloramphenicol and grown for 14 hours. A total of 40 pools were created for PCR screening. Screening was carried out as described previously [24]. Primers used for PCR screening are shown in S1 Table. Probes used for hybridization are shown in S1 Table. BAC DNA was isolated by alkaline lysis, digested with DNase-free RNase A (Sigma-Aldrich, St. Louis MO) and RNase ONE (Promega, Madison WI), followed by phenol extraction and ethanol precipitation.

BAC clones were sequenced by Sanger and Ion Torrent sequencing. For Sanger sequencing, BigDye sequencing reagents were obtained from ThermoFisher (www.thermofisher.com) and used according to the manufacturer’s instructions. Ion Torrent sequencing was carried out by the University of Wisconsin Biotechnology Center. DNA sequences were visualized using Finch TV (Geospiza, www.geospiza.com). Ion Torrent sequence reads were first aligned to the mm10 reference using TMAP aligner from the Torrent Suite software. Samtools mpileup (http://samtools.sourceforge.net/) was used to generate consensus contigs from the re-sequencing alignments and the consensus contigs were then combined with the raw fastq reads and assembled using the Velvet assembler [26, 27]. Nextgen and Sanger sequences were aligned using the program Seqman (DNASTAR, Madison WI).

### Genotyping and PCR

Tail DNA was isolated using the Puregene reagent according to the manufacturer’s instructions (Gentra, Minneapolis MN) and stored in 10 mM Tris, pH 8.0, 0.1 mM EDTA. PCR primers were designed using Primer3 software [28, 29]. Oligonucleotides were obtained from Integrated DNA Technologies (Coralville IA). Genotyping was carried out by PCR. All primer sequences and their predicted product sizes are listed in S1 Table. All PCR reactions contained 10 mM Tris, pH 9, 50 mM KCl, 0.1% Triton X100, 1.25 mM MgCl_2_, 0.1 mM dNTPs, 0.2 μM of each primer, 2.5 units *Taq* polymerase (Promega), and 25 ng tail DNA. Cycling conditions were 94°, 2 min; [94°, 30 sec; 58°, 40 sec; 72°, 1 min] for 40 cycles; 72°, 5 min. Products were separated by agarose gel electrophoresis. Predicted sizes for each genotype are listed in S1 Table. PPCD1 genotyping reactions contained the primers 6330Int19A and Sstr5, to amplify the mutant allele, and Csrp_6R and Csrp_7L, to amplify the wildtype allele. *Csrp2bp* ^*tm1a(KOMP)Wtsi*^ genotyping reactions contained the primers Csrp35277F,Csrp35277R, and Cas_R1_Term. *Csrp2bp*^*Ex*^ genotyping reactions contained the primers Csrp35277F, and mCsrpfxR. Genotyping of *Dzank1* ^*tm1a(KOMP)Mbp*^ was carried out using the primers CSD-neoF and CSD-Dzank1-ttR, to amplify the floxed allele. The wildtype allele was amplified with the primers CSD-Dzank1-F and CSD-Dzank1-ttR. The *Dzank1*^*Ex*^ allele was genotyped with the primers CSD-Dzank1-F and CSD-Dzank1-R. Cycling conditions for *Dzank1* genotyping were 94°, 5 min; [94°, 15 sec; 65°, 30 sec; 72°, 40 sec] for 10 cycles with 1° decrease/cycle; [94°, 15 sec; 55°, 30 sec; 72°, 40 sec] for 30 cycles; 72°, 5 min.

PCR amplification of sequences across the 5’-breakpoint was carried out using primers listed in S1 Table and the cycling conditions 94°, 2 min; [94°, 30 sec; 50°, 40 sec; 72°, 1 min] for 40 cycles; 72°, 5 min. PCR products were treated with Exonuclease I (Promega) and Shrimp Alkaline phosphatase (Promega) and sequenced by Sanger or cloned into pGEMTEasy (Promega, Madison WI). DNA from individual clones was isolated using the FastPlasmid Miniprep kit (5 Prime, www.5prime.com) and sequenced by Sanger sequencing.

#### Inverse PCR

Inverse PCR was carried out as described by Williams et al [30], using *Sau3A* partial digestion of PPCD1 DNA followed by circularization and amplification with primers flanking the breakpoints identified by the prior Roche-Nimblegen CGH analysis. Briefly, 0.6 μg PPCD1 genomic DNA was partially digested with *Sau3A* (New England Biolabs, Ipswich MA) and ligated with T4 DNA ligase (Promega, Madison WI) at a concentration of 3 ng/μl. PCR primers were designed using Primer3 software. Oligonucleotides were obtained from Integrated DNA Technologies (Coralville IA). PCR was carried out with High Fidelity Polymerase (Roche, www.lifescience.roche.com) according to the manufacturer’s recommendations. Cycling conditions were 94°, 2 min; [94°, 30 sec; 62°, 40 sec; 72°, 1 min] for 40 cycles; 72°, 5 min. PCR products were cloned into pGEMTEasy (Promega, Madison WI). DNA from individual clones was isolated using the FastPlasmid Miniprep kit (5 Prime, www.5prime.com) and sequenced by Sanger sequencing. Breakpoints were identified by divergence from the wildtype genomic sequence at a position other than a *Sau3A* restriction site.

### Copy number analysis

Taqman copy number analysis was carried out using the Taqman Copy Number Assays Mm0054041_cn and Mm0005227_cn, specific for *Csrp2bp* Exon 11 and *Csrp2bp* Exon 5, respectively, and the Taqman Copy Number Reference Assay (Life Technologies). Reactions were carried out in a final volume of 10 μl, containing 10 ng genomic DNA, 5 μl TaqMan Genotyping Master Mix, 0.5 μl Copy Number Assay (Mm0054041_cn or Mm0005227_cn), 0.5 μl Copy Number Reference Assay, and 2 μl distilled water. Reactions were carried out in quadruplicate. Reactions were carried out on an ABI 7900 realtime PCR machine according to the manufacturer’s recommendations. Digital PCR was carried out using ddPCR Supermix for probes, no dUTP (Bio-Rad), in a Bio-Rad QX200 Droplet Digital PCR System, according to the manufacturer’s instructions, with primer-probe sets specific for *Dzank1* Exon 20 (FAM-labelled) or *Vsx1* (HEX labelled). DNA was digested with *Mse1* prior to PCR to ensure separation of the primer binding sites. Copy number analysis using the Quantstudio 3D digital PCR system (ThermoFisher, thermofisher.com) was carried out in a volume of 15 μl, containing 32 ng genomic DNA, 7.5 μl Genotyping Master Mix, 0.8 μl gene-specific primer-probe set (Taqman Copy Number Assays Mm0054041_cn or Mm0005227_cn) and 0.8 μl reference primer-probe set (Taqman Copy Number Reference Assay).

### Gene expression

Immediately after CO_2_ euthanasia, eyes were enucleated and placed in RNAlater. Total RNA was isolated using the Qiagen RNeasy Mini kit (Qiagen, www.qiagen.com). First strand cDNA synthesis was carried out using the Bio-Rad Iscript cDNA synthesis kit (Bio-Rad, Hercules CA), according to the manufacturer’s instructions and using the supplied mixture of oligo dT and random hexamer primers. 5’-nuclease primer pairs and probes for *Csrp2bp* and *Dzank1* were designed and synthesized by Integrated DNA Technologies. 5’-nuclease qPCR was carried out in a volume of 10 μl, containing 1 μl cDNA, 0.5 μl 20X probe, 5 μl Universal Master Mix (Life Technologies, www.lifetechnologies.com), and 3.5 μl distilled water. *Ovol2* gene expression was assayed using the Mm00459038_m1 Taqman Gene Expression Assay and Taqman Gene Expression Master Mix (thermofisher.com), according to the manufacturer’s instructions. SYBR Green qPCR was carried out in a volume of 10 μl, containing 1 μl cDNA, 1 μM forward and reverse primers, 3 μl distilled water, and 5 μl Fast Start SYBR Green Master Mix (Roche, www.lifescience.roche.com). Reactions were carried out on an ABI 7900 realtime PCR machine. At the completion of each SYBR Green run, reaction products were analyzed on a 1% agarose gel to confirm the presence of a single reaction product of the expected size.

#### 3’-RACE

3’-RACE was carried out using the GeneRacer kit (Invitrogen, Carlsbad CA) according to the manufacturer’s instructions. PCR was carried out using *Pfu* Turbo polymerase (Agilent Technologies, Santa Clara CA). Reverse and nested primers are listed in S1 Table. PCR products were cloned into the vector pGEMTEasy (Promega). DNA from individual clones was isolated using the FastPlasmid Miniprep kit and sequenced by Sanger sequencing. Electrophoresis was carried out at the DNA Sequencing Facility, University of Wisconsin Biotechnology Center.

### Histology

For histological characterization, animals were sacrificed by CO_2_ inhalation and enucleated eyes fixed overnight in 10% (v/v) formalin in phosphate-buffered saline, pH 7.2, dehydrated in graded ethanol, and processed for routine paraffin embedded light microscopy. Sections were stained with hematoxylin and eosin (H&E) or by immunohistochemistry. The primary antibody used was anti-human cytokeratin, clone AE1/AE3, (DAKO, Carpenteria CA), at a dilution of 1:750. Primary antibody was omitted from negative controls. Following incubations with primary antibodies, sections were incubated with biotinylated goat anti-rabbit secondary antibody and avidin-biotinylated peroxidase complex using the Vectastain ABC kit and ImmPACT NovaRed or AMEC Red substrate according to the manufacturer’s instructions. (Vector Laboratories, Burlingame CA).

Eyes were stained for β-galactosidase activity as described by Hogan et al. [31] After staining, eyes were dehydrated in graded ethanol and processed for paraffin embedding. Sections were counterstained with Nuclear Fast Red. For preparation of frozen sections, newborn animals were sacrificed by CO_2_ inhalation followed by cervical dislocation. Heads were frozen in OCT compound and sectioned in a cryostat, followed by β-galactosidase staining and photography.

## Results

### Gene structure of the mouse *Ppcd1* locus

**Fig 1:**
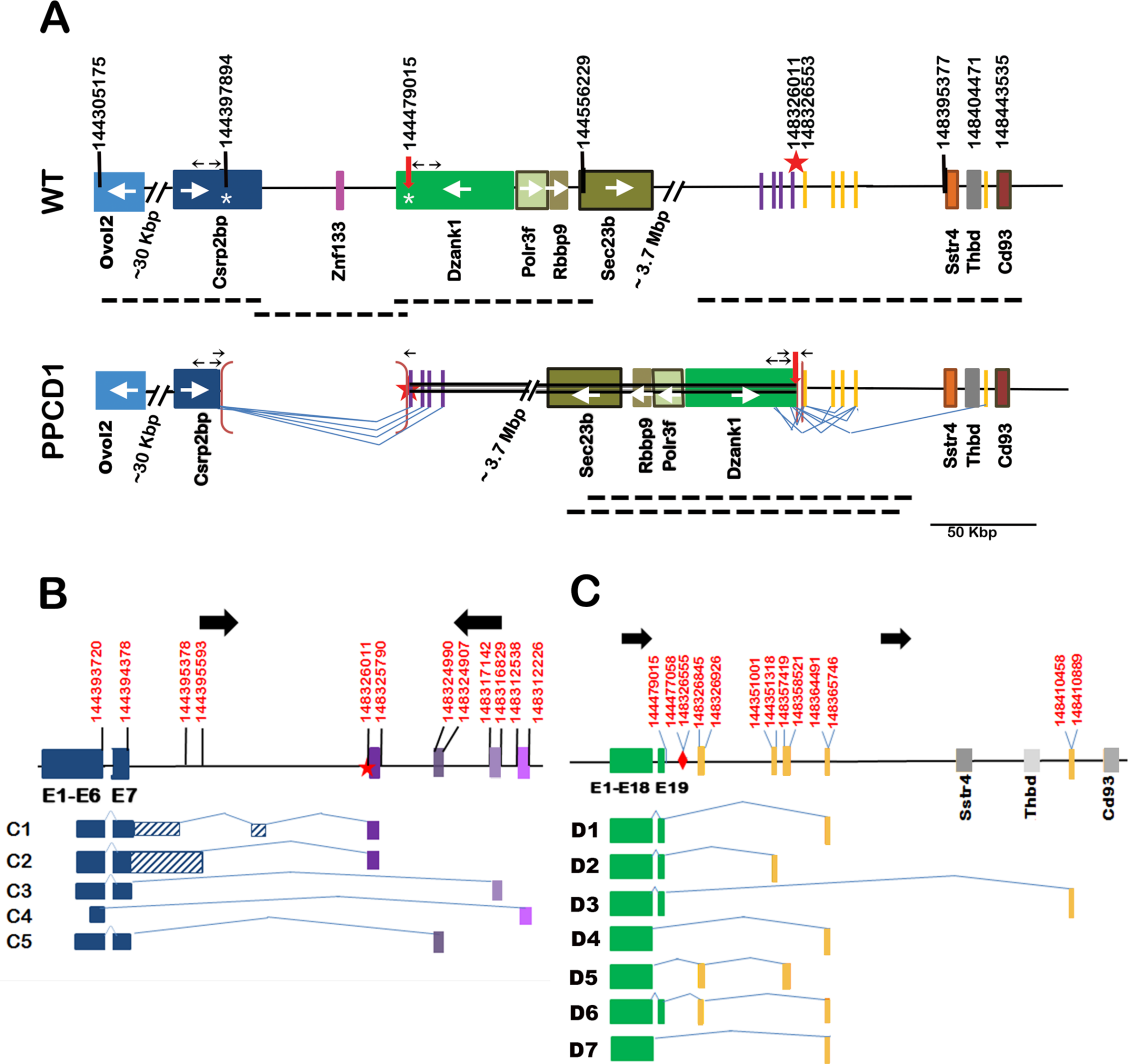
Structure of the *Ppcd1* gene rearrangement and alternative transcripts. (A) Structure of the *Ppcd1* mutant gene. The wildtype map, displaying the region from *Ovol2* to *Cd93*, is shown on top and the mouse *Ppcd1* map below. Coordinates are shown at the top. Solid boxes indicate genes (labelled) or sequence blocks. Arrows inside gene boxes indicate the direction of transcription. The heavy arrow and star at positions 144479015 and 148326011 indicate the end points of the inverted segment. The inverted region is indicated by a double line. Brackets indicate deletions. The endpoints of the 81121 bp deletion are shown on the wildtype chromosome as white asterisks, at positions 144397894 and 144479015, in the *Csrp2bp* and *Dzank1* genes. Heavy dotted lines below each map indicate positions of BAC clones. Thin lines indicate splice patterns. Small horizontal arrows indicate the positions of primers. Arrows pointing away from each other indicate primers used for inverse PCR and arrows pointing toward each other indicate primers used to confirm deletion endpoints. (B) *Csrp2bp* fusion transcripts. *Csrp2bp* exons are indicated by dark blue solid blocks. Purple blocks indicate positions of downstream sequences spliced to *Csrp2bp* Exons 6 or 7. Striped blocks indicate intron sequences. Lines indicate splice patterns. Coordinates (GRCm38) are indicated at the top. Heavy black arrows indicate chromosomal orientation. The star indicates the breakpoint of the inversion. (C) *Dzank1* fusion transcripts. *Dzank1* exons are shown in green. Gray blocks indicate positions of genes. Yellow blocks indicate positions of downstream sequences spliced to *Dzank1* Exons 18 or 19. Lines indicate splice patterns. Coordinates (GRCm38) are indicated at the top. Heavy black arrows indicate chromosomal orientation. The diamond indicates the breakpoint of the inversion.

The *Ppcd1* allele has been backcrossed to DBA/2J (D2) for greater than 27 generations. Heterozygotes exhibit the mouse PPCD1 phenotype described previously [20] and homozygotes do not survive early gestation. Thus, characterization of genomic rearrangements was performed using DNA from viable heterozygous animals. Our previous mapping studies localized the mouse *Ppcd1* locus to mouse Chromosome 2, between the markers D2Mit259 and D2Mit282, located at 142.8 Mbp and 148.9 Mbp, respectively on the GRCm38 reference genome. Roche Nimblegen Comparative Genome Hybridization (CGH) service had previously identified the endpoints of a putative 87 Kbp duplication in this region. We have now fully characterized the gene rearrangements in this region by a combination of PCR analysis and BAC cloning and show that the endpoints identified by CGH analysis are actually the breakpoints of a 3.9 Mbp chromosomal inversion and the putative duplication is actually a deletion. The wildtype and mutant *Ppcd1* alleles are shown in Fig 1A.

The mutant *Ppcd1* allele consists of a 3.9 Mbp inversion flanked by 81 Kbp and 542 bp deletions at the proximal (5’) and distal (3’) breakpoints (Fig 1A). Note that all coordinates in this manuscript refer to GRCm38 (the 87 Kbp deletion size previously reported is based on GRCm37). The *Ppcd1* rearrangement leads to deletion of the 3’-termini of the genes *Csrp2bp* and *Dzank1* and the entire *Znf133* pseudogene, concomitant with introduction of novel downstream genomic sequences adjacent to *Csrp2bp* Exon 7 and *Dzank1* Exon 19. The proximal breakpoint of the inversion is a fusion between Chr2:144397894 bp, located in Intron 7 of *Csrp2bp*, and Chr2:148326011 bp, located in an intergenic region 69366 bp upstream of the gene *Sstr4*. The distal breakpoint of the inversion is a fusion between Chr2:144479015 bp, located in Intron 19 of *Dzank1*, and Chr2:148326553 bp, located 68824 bp 5’ of the gene *Sstr4*. Sequences adjacent to each breakpoint, Chr2:144397894 to 144479015 and Chr2:148326011 to 148326553, are deleted. The endpoints of the larger, 81121 bp (i.e., 81 Kbp) deletion are located in *Csrp2bp* Intron7 and *Dzank1* Intron 19; thus Exons 8 through 11 of *Csrp2bp*, Exons 20 and 21 of *Dzank1*, and the entire *Znf133* gene are deleted.

The breakpoints of the gene rearrangement were identified by inverse PCR of PPCD1 genomic DNA. The distal breakpoint of the rearrangement was identified by inverse PCR with the primers INV20L/INV20R, which yielded two sequences, one corresponding to the expected wildtype sequence and one containing *Dzank1* Intron 19 sequences, in the reverse orientation (*Dzank1* is transcribed on the minus strand), followed by sequences located ~69 Kbp 5’ of the gene *Sstr4*, in the forward orientation. A 542 bp sequence between Chr2:148326011 and Chr2:148326553 is deleted. Fusion of *Dzank1* Intron 19 sequences to downstream sequences was confirmed by PCR analysis using the primer pair 6330Int19/Sstr7, which amplified a 221 bp fragment in PPCD1 but not wildtype animals (S1 Fig). S2 Fig shows the alignment of sequences spanning the distal breakpoint with the mouse reference sequence (GRCm38).

The proximal breakpoint was also identified by inverse PCR. The primers INV_5L and INV_5R yielded an 830 bp fragment corresponding to the expected wildtype sequence, as well as an amplicon that contained *Csrp2bp* Intron 7 sequence in the forward orientation fused to a sequence located ~69 Kbp 5’ of the gene *Sstr4*, in the reverse orientation. PCR amplification of a 239 bp fragment with primers CRIT28854 and SSTR7 in DNA from PPCD1 but not wildtype animals confirmed fusion of *Csrp2bp* Intron 7 sequences to the predicted downstream sequences (S3 Fig). The location of the *Csrp2bp* Intron 7 breakpoint was further confirmed by PCR amplification with a series of primer pairs spanning the breakpoint (S4 Fig). S5 Fig shows alignment of the *Ppcd1* assembly sequence spanning the proximal breakpoint with the genomic sequences from Csrp2bp Intron 7 from D2 (shown in S6 Fig) and the reference genome GRCm38.

Two Short Interspersed Nuclear Elements (SINE) are located near the breakpoints. A 181 bp B2_Mm1a element, present in wildtype D2 DNA and the Celera genome, but not B6, is located in *Csrp2bp* Intron 7, 111 bp 5’ of the proximal breakpoint. A PB1D9 SINE element is located in *Dzank1* Intron 19, ~200 bp from the distal breakpoint, in both the D2 and B6 genomes.

Further confirmation of the distal breakpoint was obtained by isolation and sequencing of a BAC clone spanning the distal breakpoint (Fig 1A). Screening of a BAC library constructed from PPCD1 DNA with probes specific for *Csrp2bp* Exons 7, 9, or 11, or *Dzank1* Exons 20 or 21 yielded overlapping clones spanning the wildtype chromosome from Ch2:144286355 to Chr2:14471022, containing the genes *Ovol2*, *Csrp2bp*, *Znf133*, *Dzank1*, *Polr3f*, *Rbbp9*, *and Sec23B*. Using a probe spanning the distal breakpoint, we obtained one wildtype clone, extending from Chr2:148286314 to Chr2:148444495 and containing the genes *Sstr4* and *Thbd*, and two mutant clones spanning the distal breakpoint, containing *Sec23B*, *Rbbp9*, *Polr3f*, *Dzank1* to the breakpoint, and sequences distal to the breakpoint. Sanger and Ion Torrent sequencing of the mutant clones confirmed that the breakpoint was indeed that identified by PCR and consistent with the gene structure shown in Fig 1A. The sequence of the mutant BAC clone has been deposited in GenBank with the accession number KU729003. Screening of the BAC library with probes spanning the proximal breakpoint or probes derived from *Csrp2bp* Exon 7 yielded only wildtype or nonspecific clones. No clones corresponding to any other rearrangements were found with any of the probes.

Alignment of the sequences spanning the breakpoints indicated that sequences adjacent to each breakpoint, Chr2:144397894 to 144479015 and Chr2:148326011 to 148326553, are deleted. Although the endpoints of the 81 Kbp deletion correspond very closely to the endpoints of the sequence previously identified as duplicated by Nimblegen CGH analysis, the data are consistent with hemizygous deletion, not duplication, of this sequence. To resolve the copy number discrepancy, the copy number of the 81 Kbp segment was confirmed by three independent copy number analyses (Table 1). Taqman copy number analysis was carried out using the probes from *Csrp2bp* Exon 5, located outside the 81 Kbp segment, and *Csrp2bp* Exon 11, located inside the segment. We found that two copies of *Csrp2bp* Exon 5 are present in both wildtype and PPCD1 DNA. However, the copy number of *Csrp2bp* Exon 11 is two in wildtype DNA and only one in PPCD1 DNA, consistent with deletion of sequences distal to Exon 7 of *Csrp2bp*. Similar results were obtained with digital PCR using probes for *Vsx1*, located just outside the mapped PPCD1 critical region, and *Csrp2bp* Exon 11 and *Dzank1* Exon 20, both located inside the putative deletion. The copy numbers of both *Csrp2bp* Exon 11 and *Dzank1* Exon 20 were found to be 2 in wildtype DNA and 1 in PPCD1 DNA using two different digital PCR platforms. The precision of the digital PCR is sufficient to distinguish between 1 and 1.5 copies and confirms that the 81 Kbp segment is indeed deleted rather than duplicated. The Nimblegen CGH service is no longer in existence and it is not possible to further examine the basis for the incorrect copy number assignment.

**Table 1:** Determination of PPCD1 copy number.

Genotype	Copy Number			
	Csrp2bp Exon 5^a^	Csrp2bp Exon 11^a^	Dzank1 Exon 20^b^	Csrp2bp Exon 11^c^
WT	2.0 ± 0.32 (4)	2.00 ± 0.2 (4)	2.01 ± 0.02(5)	1.92 (2)
PPCD1	1.94 ± 0.13 (4)	1.04 ± 0.03 (4)*	1.02 ± 0.02 (5)*	0.98 (2)

Copy number was determined using Taqman Copy Number qPCR^a^

Bio-Rad Digital PCR^b^, or

ABI Digital PCR^c^.

Copy numbers determined by Taqman qPCR and ABI Digital PCR are expressed relative to the Life Technologies Copy Number Reference. Bio-Rad Digital PCR values are expressed relative to the copy number of *Vsx1*. Values are expressed as mean +/- SD (number of animals tested).

* indicates significant difference from WT, P < 0.0001.

### Fusion transcripts produced by the PPCD1 Inversion

The *Ppcd1* inversion translocates sequences normally located in an intergenic region, approximately 3.9 Mbp 3’ of *Csrp2bp* and 70 Kbp 5’ of the gene *Sstr4*, to base 3748 of *Csrp2bp* Intron 7. In addition to wildtype *Csrp2bp* transcripts, 5 alternative fusion transcripts involving *Csrp2bp* were detected by 3’-RACE. Novel *Csrp2bp* fusion transcripts produced as a result of the *Ppcd1* rearrangement are shown in Fig 1B. The *Csrp2bp* C1 transcript contains Exons 1 through 7 followed by Intron 7 bases 1 through 992. This sequence is then spliced to Intron 7, bases 3662 to 3748, in the forward orientation, followed by the sequence Chr2:148326011 to 148325790, in the inverse orientation. The *Csrp2bp* C2 transcript contains Exons 1 through 7 followed by 1209 bp of Intron 7 sequence spliced to the same downstream sequence found in the C1 transcript. No known coding sequences are located in this region. Termination codons are located 112 bp past Exon 7, leading to, at most, addition of 37 extra amino acids. These amino acids do not contain any known protein domain structure. Two transcripts where Exon 7 is directly spliced to downstream sequences (C3 and C5) and one where Exon 6 is spliced directly to downstream sequences (C4), were also identified.

The inversion event also fuses genomic sequence beginning at Chr2:144326553, 30487 bp 5’ of the gene *Sstr4*, to base 1966 of *Dzank1* Intron 19. Fig 1C shows the structures of 7 *Dzank1* fusion transcripts arising from the PPCD1 inversion. Splicing occurs from either Exon 18 or Exon 19 of *Dzank1* to the downstream sequences. The *Dzank1* D1 through D4 and D7 transcripts are each the product of a single splice event from *Dzank1* Exon 18 or 19 to a single downstream sequence. In the case of *Dzank1* D5 and D6 transcripts, two splice events were observed, to generate the equivalent of two additional exons fused to *Dzank1*. All the novel sequences originate upstream of the *Sstr4* start site, or, in one case, in the intergenic region between the genes *Thbd* and *Cd93*.

#### Expression of normal and fusion transcripts

**Fig 2:**
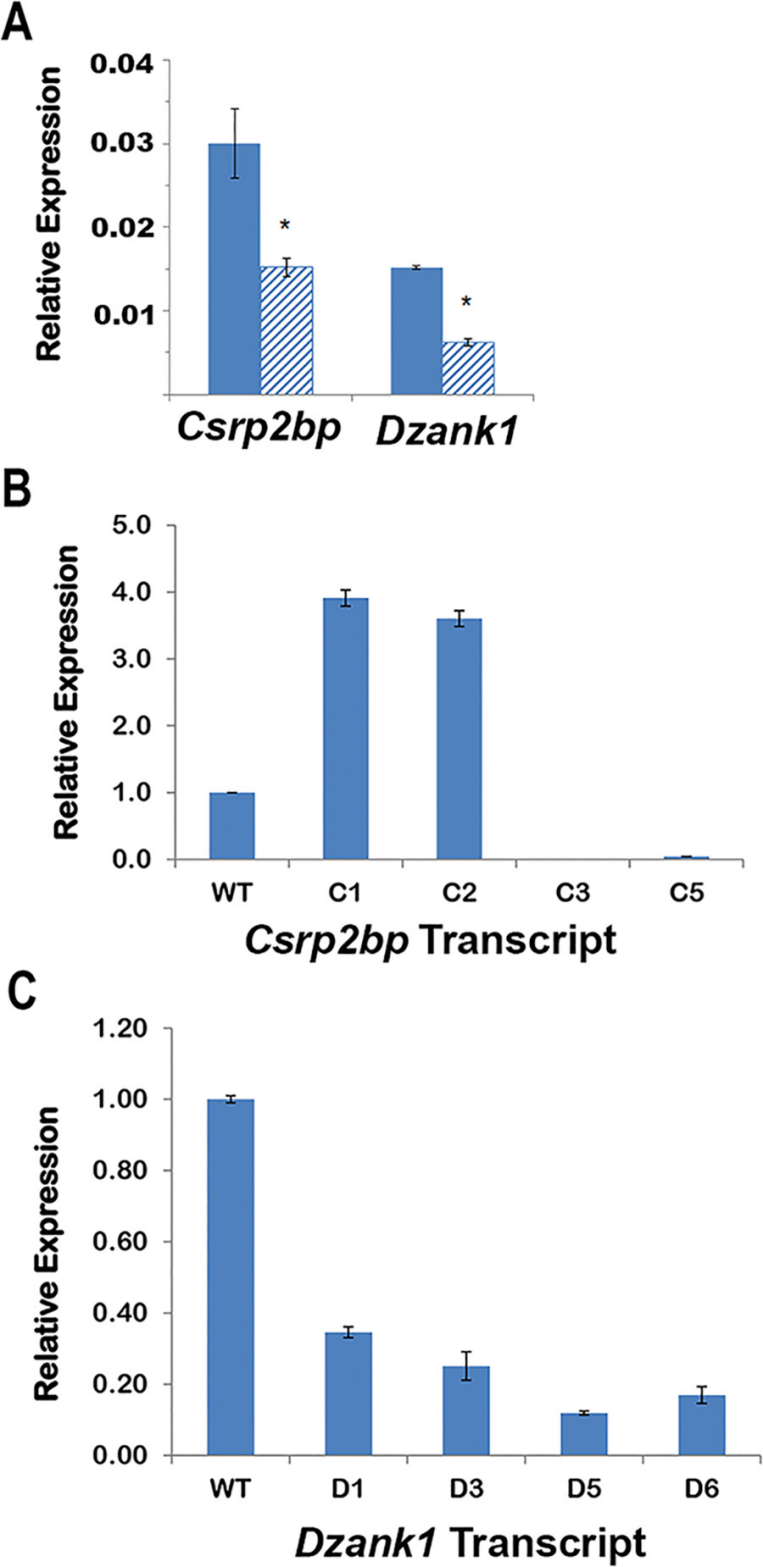
qPCR of *Csrp2bp* and *Dzank1* transcripts. (A) Expression levels of *Csrp2bp* and *Dzank1* wildtype transcripts. Solid bars are wildtype and striped bars indicate PPCD1. Values are expressed relative to *Gapdh*. * indicates P < 0.05. (B) Expression of *Csrp2bp* fusion transcripts. Values are expressed relative to the expression level of the wildtype transcript. (C) Expression of *Dzank1* fusion transcripts. Values are expressed relative to the expression level of the wildtype transcript. All values are mean ± SD, n = 3.

Previous qPCR results [20] demonstrated decreased expression of *Csrp2bp* Exons 9 and 10 in PPCD1 eyes, which is consistent with the observed gene rearrangement. To investigate expression levels of fusion transcripts relative to expression levels of the wildtype transcript, qPCR primer pairs specific for the wildtype or the novel transcripts were designed (S1 Table). In agreement with previous qPCR results, levels of the wildtype *Csrp2bp* and *Dzank1* transcripts (i.e., those containing *Csrp2bp* Exons 7 and 8 and *Dzank1* Exons 19 and 20) in PPCD1 eyes are approximately half that observed in wildtype eyes (Fig 2A). In wildtype eyes, expression levels of *Csrp2bp* fusion transcripts ranges from undetectable to less than 0.1% of the wildtype transcript. In PPCD1 eyes, *Csrp2bp* transcripts C1, C2, C3, and C5 are expressed at levels readily detectable by qPCR. Fig 2B shows that the *Csrp2bp* C1 and C2 transcripts are expressed at levels nearly 4 times that of the wildtype *Csrp2bp* transcript. *Csrp2bp* C3 and C5 transcripts are also detectable at low levels (< 5% of wildtype) in PPCD1 eyes.

Expression levels of *Dzank1* fusion transcripts in wildtype eyes are also less than 0.1% of the wildtype *Dzank1* transcript. Four *Dzank1* fusion transcripts (D1, D3, D5, and D6) are detectable by qPCR. Fig 2C shows that these are expressed in PPCD1 eyes at levels ranging from 12% to 37% of the *Dzank1* wildtype transcript. PCR amplification of the remaining transcripts (*Csrp2bp* C4 and *Dzank1* D2, D4, and D7), in which much of the sequence fused to *Csrp2bp* or *Dzank1* was repetitive, yielded nonspecific products and were not further evaluated.

### *Csrp2bp* and *Dzank1* expression patterns

To examine the sites of expression of *Csrp2bp* and *Dzank1*, we employed “Knockout First” mouse models, which carry a *β-Gal-neo* cassette [32] expressing β-galactosidase (*LacZ*) activity under control of either the *Csrp2bp* or *Dzank1* promoter. The *Csrp2bp* ^*tm1a(KOMP)Wtsi*^ allele, generated by the Wellcome Trust Sanger Institute and hereafter referred to as *Csrp2bp*^*tm1a*^, carries a *β-Gal-neo* cassette in Intron 2 that disrupts the *Csrp2bp* coding sequence and allows identification of the *Csrp2bp*-expressing tissues, plus *loxP* sites flanking Exon 3 allowing Cre-mediated excision. For details, see http://www.mousephenotype.org/data/alleles/project_id?ikmc_project_id=38090. Similarly, the *Dzank1* ^*tm1a(KOMP)Mbp*^ (*Dzank1*^*tm1a*^) allele carries a *β-Gal*-*neo* cassette in Intron 3 and *loxP* sites flanking Exon 4 (https://www.komp.org/ProductSheet.php?cloneID=663824).

**Fig 3:**
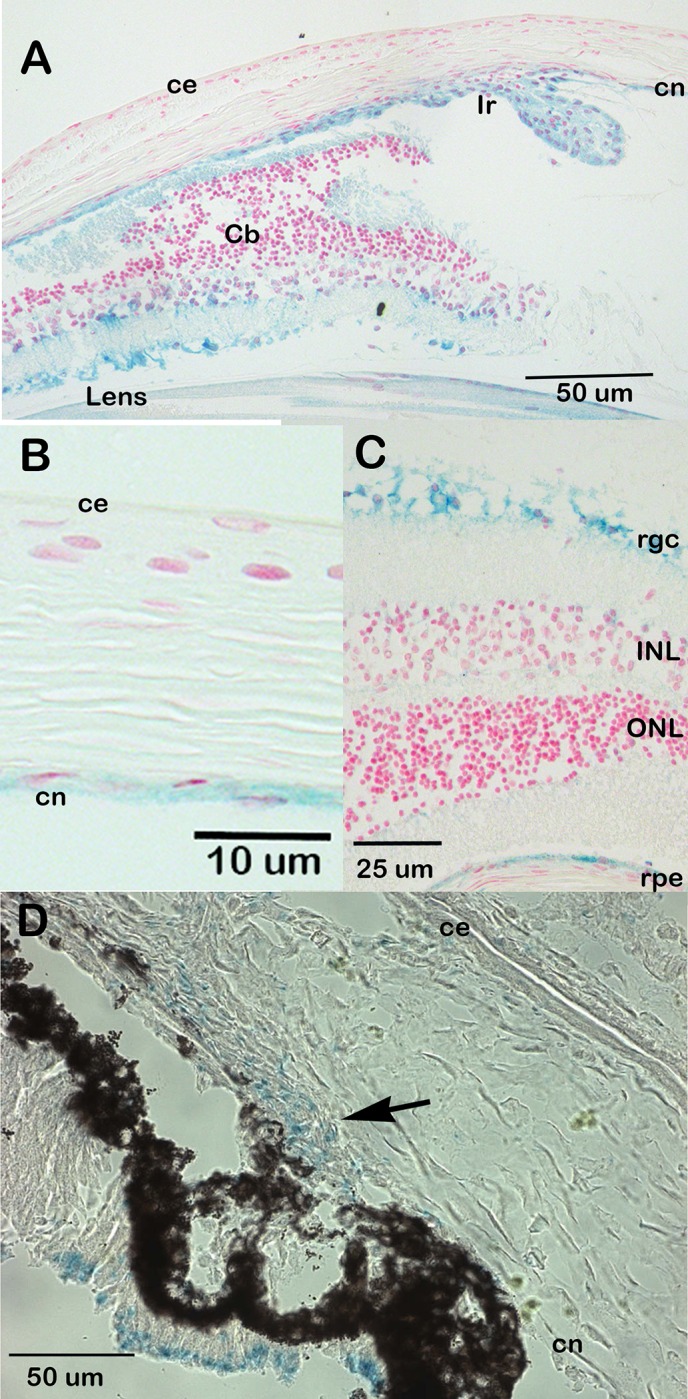
Expression of *Csrp2bp* in the eye. Blue staining represents *Csrp2bp* expression. (A) Adult (3 months) iridocorneal angle, B6/albino background. ce, corneal epithelium; cn, corneal endothelium; Ir, iris; Cb, ciliary body. (B) Adult central cornea (B6/albino) (C) Retina (B6/albino). rgc, retinal ganglion cells; ONL, outer nuclear layer; INL, inner nuclear layer; rpe, retinal pigment epithelium. (D) Neonatal (P0) iridocorneal angle (B6). The arrow points to the developing iridocorneal angle.

**Fig 4:**
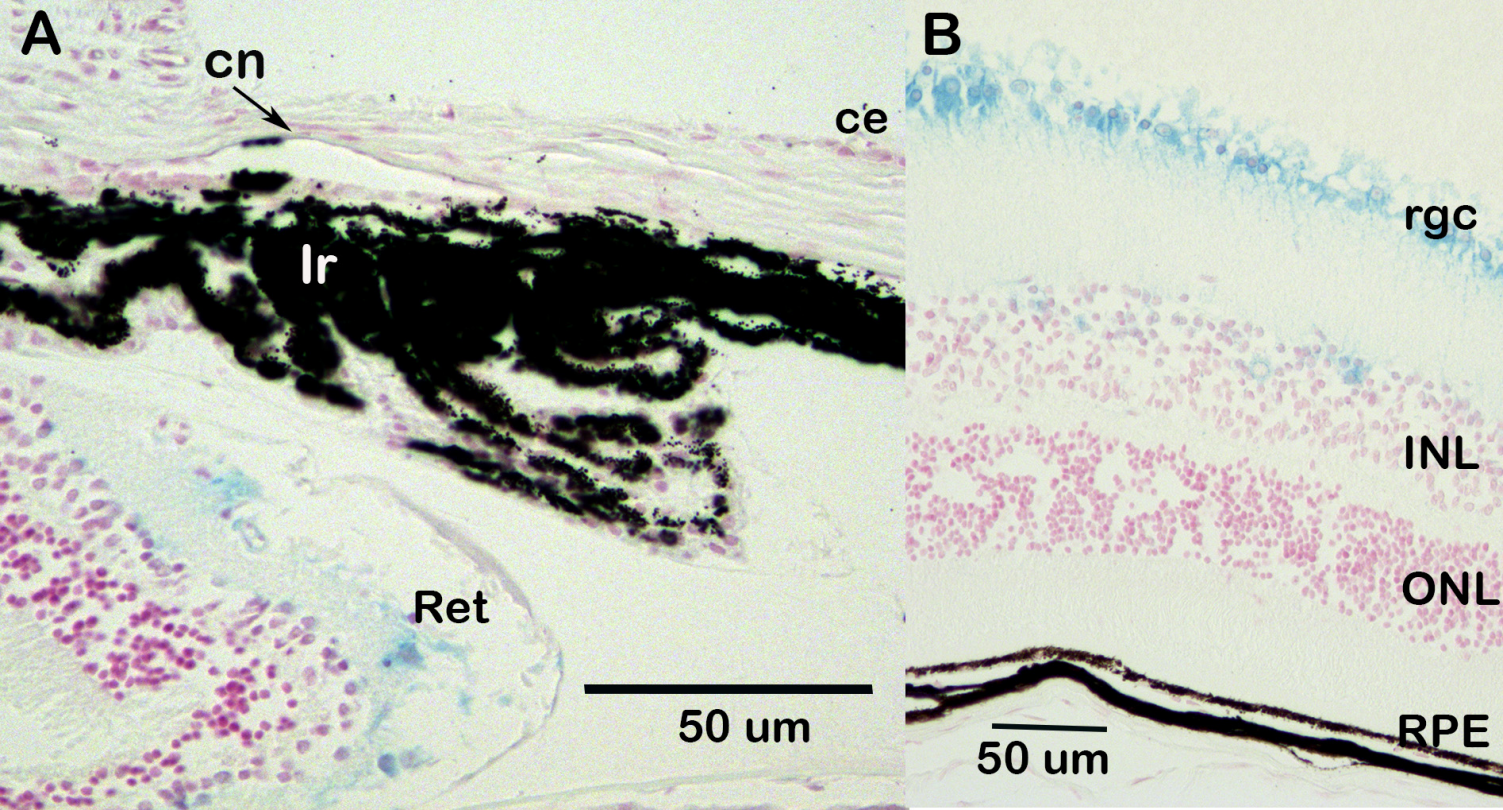
Expression of *Dzank1* in the eye. Blue staining represents *Dzank1* expression. Background is B6. Age, 1 month. (A) Iridocorneal angle. ce, corneal epithelium; cn, corneal endothelium; Ir, iris; Ret, retina. (B) Retina. rgc, retinal ganglion cell; INL, inner nuclear layer; ONL, outer nuclear layer; rpe, retinal pigment epithelium.

β-galactosidase staining of eyes from animals carrying the *Csrp2b* ^*tm1a*^ allele indicates that *Csrp2bp* is expressed at significant levels in the eye. In the adult eye, *Csrp2bp* expression was observed in the iris and ciliary body, corneal endothelium, trabecular meshwork, lens, retinal ganglion cells and retinal pigment epithelium (Fig 3A–3C). In neonatal animals, *Csrp2bp* expression was observed in the developing iridocorneal angle and corneal endothelium (Fig 3D). Ocular expression of *Dzank1* is more restricted (Fig 4). *Dzank1*-driven β-galactosidase activity was observed only in the retinal ganglion cells and inner nuclear layer. *Dzank1*-driven β-galactosidase expression was not observed in the corneal endothelium or iridocorneal angle.

### Ocular phenotypes associated with *Csrp2bp* and *Dzank1* deficiency

To test the hypothesis that haploinsufficiency of *Csrp2bp* and *Dzank*1 is responsible for mouse PPCD1, the ocular phenotypes of animals heterozygous for both genes were examined. The International Mouse Phenotyping Consortium has reported phenotypic changes for both *Csrp2bp* ^*tm1a*^ and *Dzank1* ^*tm1a*^ homozygous null animals, consistent with effective disruption of gene expression by the β-Gal-*neo* cassette (http://www.mousephenotype.org) [33]. To remove any possibility of confounding results due to disruption of expression of neighboring genes by *neo*, *Csrp2b* ^*tm1a*^/+ mice were crossed to B6N.129S4-*Gt(ROSA)26Sor*^*tm1(FLP1)Dym*^/J (ROSA flp) mice to remove the β-Gal-*neo* cassette and to B6.C-Tg(CMV-cre)_1_Cgn/J (CMV-cre) mice to delete the targeted exon. The resulting allele, in which both the β-Gal-*neo* cassette and Exon 3 of *Csrp2bp* are deleted, is designated *Csrp2bp*^*Ex*^. The *Csrp2b* ^*tm1a*^ and *Csrp2bp*^*Ex*^ alleles were each backcrossed onto the D2 background for >7 generations. No phenotypic differences were observed between the *Csrp2b* ^*tm1a*^ and *Csrp2bp*^*Ex*^ alleles.

**Fig 5:**
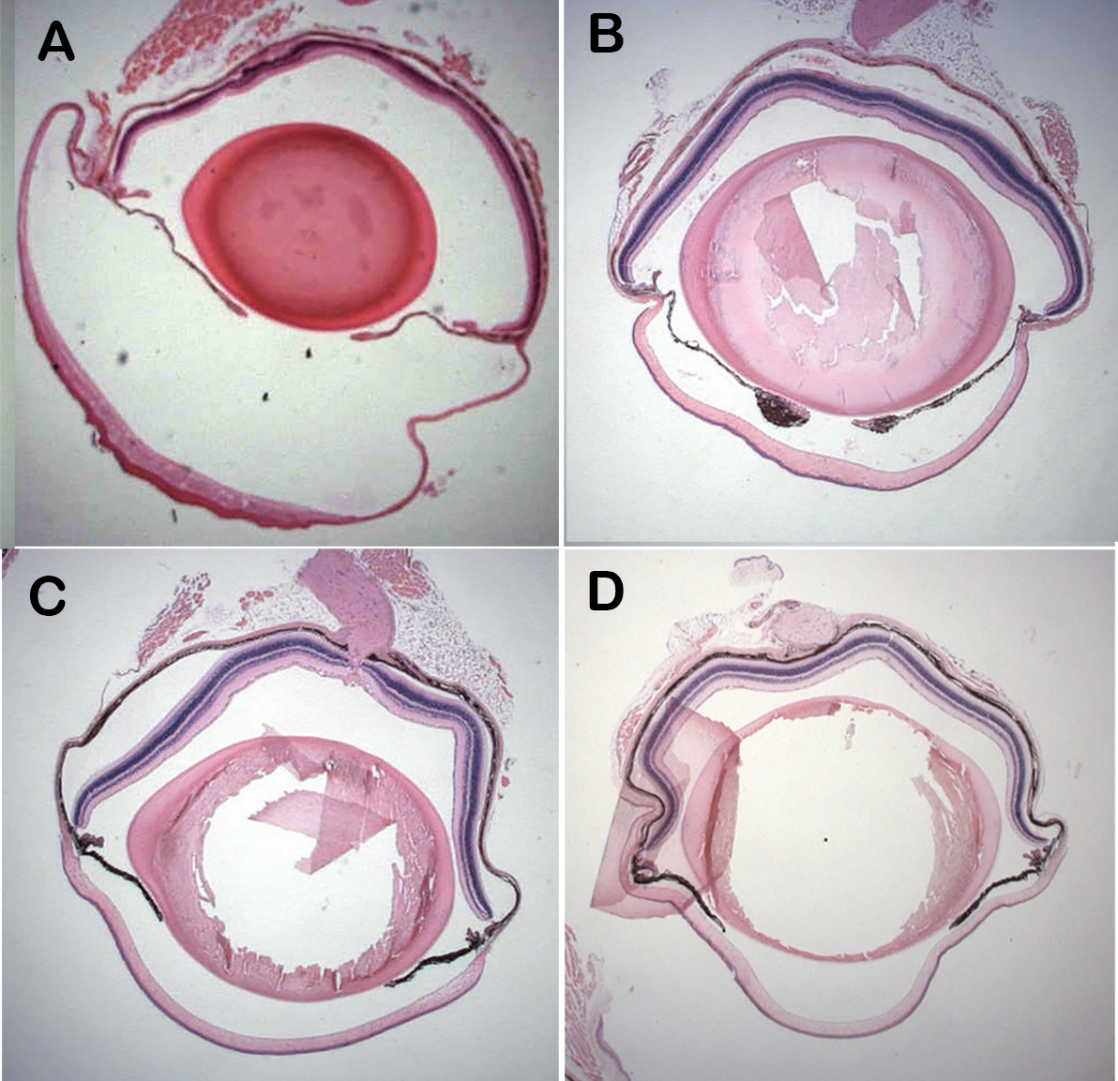
Lack of anterior chamber enlargement in *Csrp2bp*^*Ex*^/+ and *Csrp2bp*^*Ex*^/*Dzank1*^*tm1a*^ double heterozygous mice. (A) PPCD1/D2 mouse, age 3 weeks. (B) *Csrp2bp*^*Ex*^/+ mouse, age 8 months. (C) *Csrp2bp*^*Ex*^/*Dzank1*^*tm1a*^ double heterozygote, (D2xB6 F1 background), age 5 months. (D) Wildtype B6 mouse, age 5 months. Magnification 12.5X.

**Fig 6:**
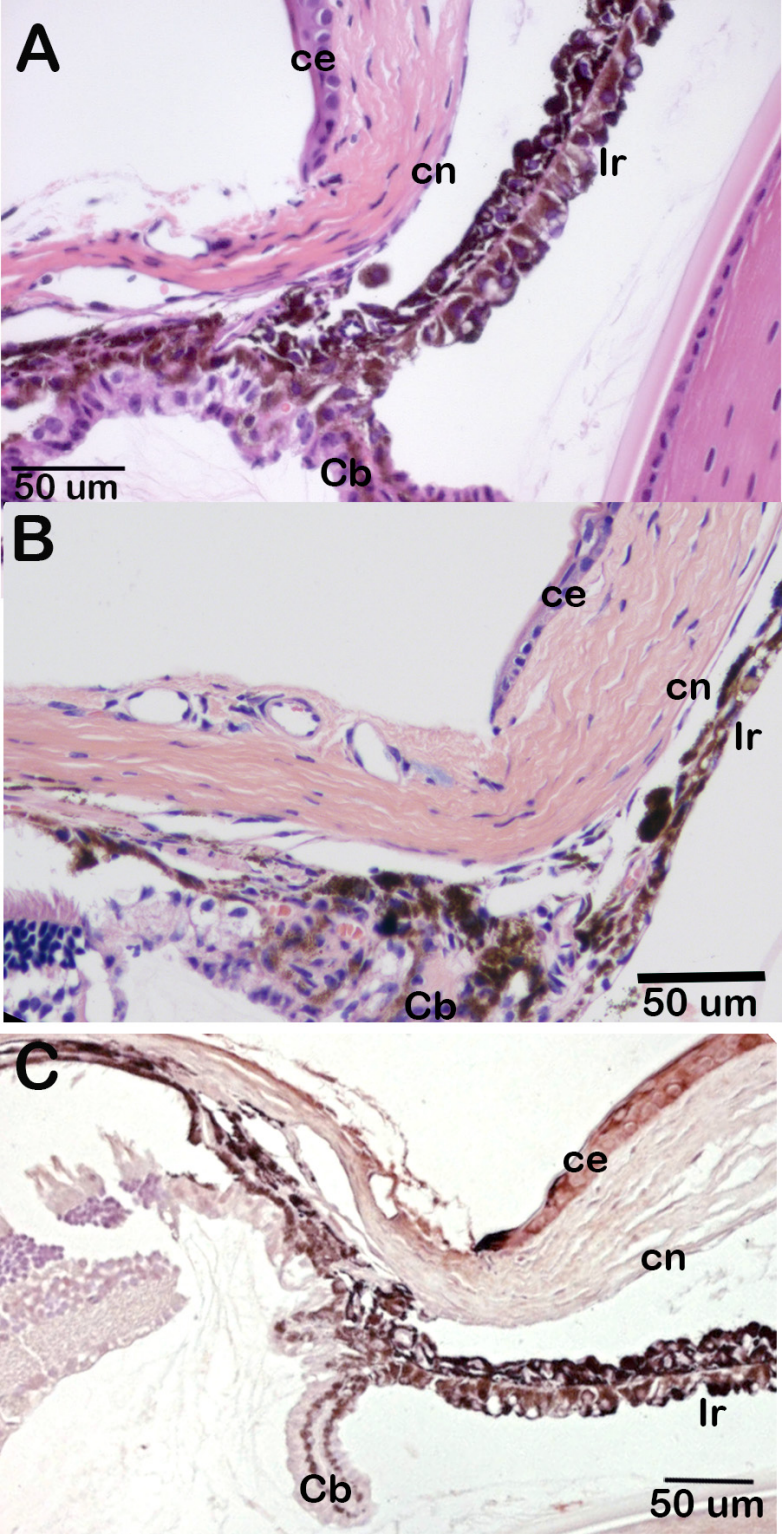
Lack of corneal endothelial cell metaplasia in *Csrp2bp* haploinsufficient mice. (A) *Csrp2bp*^*tm1a*^/+, age 3 months, H &E. (B) *Csrp2bp*^*Ex*^/+, age 8 months, H&E. (C) *Csrp2bp*^*tm1a*^/+, age 2 months, pan-cytokeratin immunohistochemistry. Ir, iris; ce, corneal epithelium; cn, corneal endothelium; Cb, ciliary body.

Eyes of *Csrp2bp*^*Ex*^/+ animals are of normal size as late as 8 months of age and the anterior chamber is not enlarged (Fig 5). Histologic examination of H&E-stained eyes did not reveal evidence of epithelized corneal endothelium at 3 or 8 months of age (Fig 6A and 6B). In contrast to the marked pan-cytokeratin immunoreactivity present as early as 3 weeks of age in PPCD1 animals on the D2 background [20], pan-cytokeratin immunoreactivity is absent in *Csrp2bp* ^*tm1a*^/+ eyes (Fig 6C).

**Fig 7:**
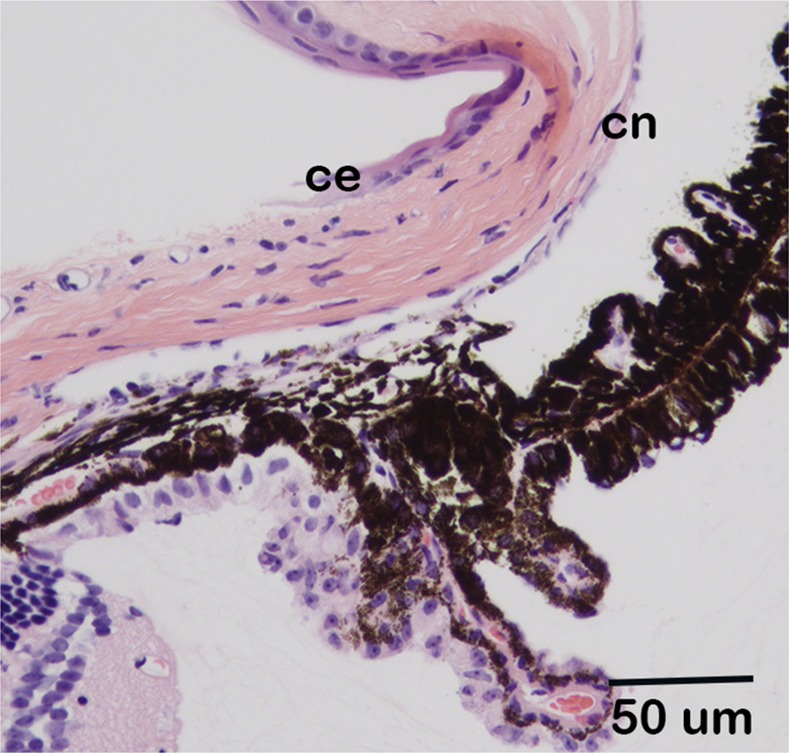
Normal appearance of the iridocorneal angle of *Csrp2bp*^*Ex*^/ *Dzank1*^*tm1a*^ double heterozygous mice. *Csrp2bp*^*Ex*^/ *Dzank1*^*tm1a*^ double heterozygote, age 5 months, H&E. ce, corneal epithelium; cn, corneal endothelium.

To determine if combined haploinsufficiency of *Csrp2bp* and *Dzank1* is responsible for mouse PPCD1, *Csrp2bp*^*Ex*^*/+* animals on the D2 background were crossed to *Dzank1* ^*tm1a*^*/+* animals on the B6 background to generate *Csrp2bp*^*Ex*^/*Dzank* ^*tm1a*^ double heterozygotes. Although development of visibly enlarged eyes is delayed on the B6 background, we have previously shown that 76% of PPCD1 animals on the D2 x B6 F1 genetic background exhibit grossly observable enlarged eyes at 3 months of age [20]. Visual examination of eyes from four 4–5 month old *Csrp2bp*^*Ex*^/*Dzank* ^*tm1a*^ double heterozygotes did not reveal visibly enlarged eyes (P < .002 compared to PPCD1 D2 X B6 F1 animals). Lack of anterior chamber enlargement was confirmed by examination of histological sections (Fig 5C) and the iridocorneal angle appears normal (Fig 7).

### PPCD1-associated embryonic lethality is due to disruption of *Csrp2bp*

While heterozygosity of *Ppcd1* leads to the PPCD1 ocular phenotype, homozygosity of *Ppcd1* leads to embryonic lethality [20]. Crosses between *Csrp2bp*^*tm1a*^ heterozygotes yield only 10% of the expected number of *Csrp2bp* ^*tm1a*^ homozygous offspring, while *Dzank1*^*tm1a*^ homozygotes are viable (www.mousephenotyping.org) [33]. Evidence that homozygous disruption of *Csrp2bp* is responsible for embryonic lethality in PPCD1 animals comes from results of a complementation analysis. Table 2 presents the results of crosses between *Csrp2bp*^*Ex*^*/+* and *Ppcd1/+* animals, showing that the combination of the *Ppcd1* and *Csrp2bp*^*Ex*^ alleles results in embryonic lethality. Similar results were obtained using *Csrp2bp* ^*tm1a*^*/+* animals. In both cases, the presence of the wildtype *Dzank1*, *Ovol2*, and *Znf133* alleles is unable to rescue the embryonic lethality associated with the *Ppcd1* allele. In contrast, the presence of a wildtype *Csrp2bp* allele and a *Dzank1* ^*tm1a*^ allele does rescue the embryonic lethality. Rescue by the wildtype *Csrp2bp* allele was incomplete, with approximately 50% of *Ppcd1*/*Dzank1* ^*tm1a*^ double heterozygotes dying prior to birth.

**Table 2:** Complementation Analysis of PPCD1 Embryonic Lethality.

Cross	Number of Live Progeny^a^					
	*Ppcd1/*	*Ppcd1/*	*Csrp2bp^Ex or Tm1a^/*	*Ppcd1/*	*Dzank1^Tm1a^/*	+/+
	+	*Csrp2bp^Ex or Tm1a^*	+	*Dzank1^Tm1a^*	+	
^**b**^*Csrp2bp*^*Ex*^*/+* x *Ppcd1/+*	14	0	11	-	-	5
^**c**^*Csrp2bp*^*Tm1a*^*/+ x Ppcd1/+*	6	0	7	-	-	11
^**d**^*Dzank1*^*Tm1a*^*/+* x *Ppcd1/+*	24	-	-	9	18	25

^a^Genotypes are as indicated. Survival was scored at weaning. +, wildtype.

^b^P < 0.002

^c^P < 0.015

^d^P < 0.04

### *Ovol2* gene expression

**Fig 8:**
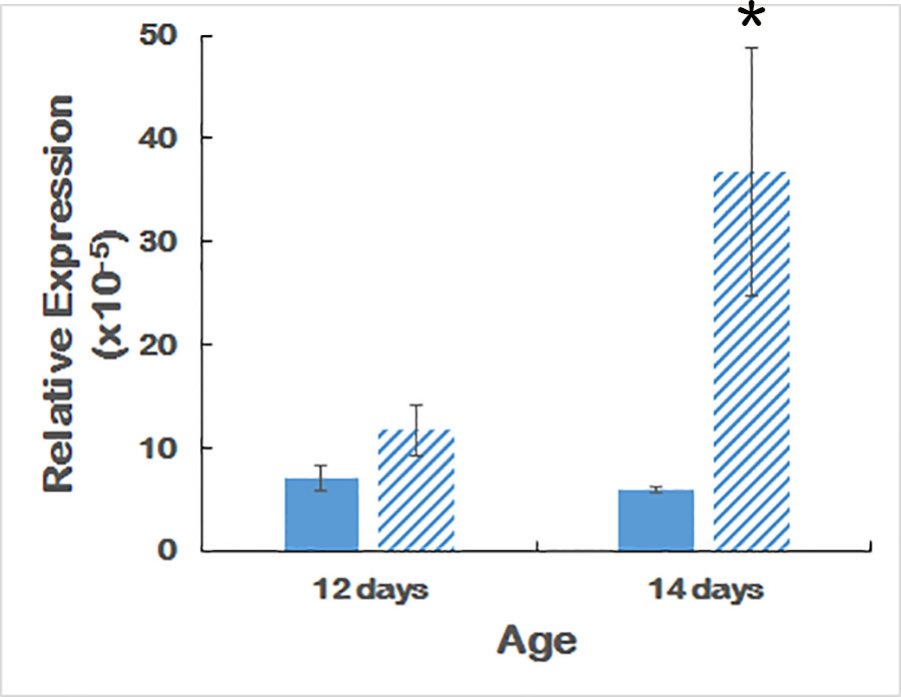
qPCR of *Ovol2*. Expression levels of *Ovol2* in wildtype and PPCD1 eyes at 12 and 14 days of age. Solid bars indicate wildtype and striped bars indicate PPCD1. Values are expressed relative to *Gapdh*. n = 6 for each genotype at 12 days and n = 3 for each genotype at 14 days. *, P = 0.05.

*Ovol2* transcripts are present at low but detectable levels in the eye. Fig 8 shows that *Ovol2* is significantly upregulated in 14-day old but not 12-day old mouse eyes.

## Discussion

We have now fully characterized the chromosomal rearrangement that causes the PPCD1 phenotype. In particular, we have now discovered that the *Ppcd1* locus contains a 3.9 Mbp chromosomal inversion coupled to 81 Kbp and 549 bp deletions. We have identified novel transcripts arising from *Csrp2bp* and/or *Dzank1*, as well as upregulation of an adjacent gene, *Ovol2* that may be responsible for corneal endothelial metaplasia. This potential disease mechanism is consistent with the *Ppcd1* chromosomal rearrangement, the autosomal dominant inheritance of PPCD1, and the phenotypes of *Csrp2bp1* and *Csrp2bp1/Dzank1* haploinsufficient animal models. Evidence against a role for *Znf133* haploinsufficiency includes the observations that this locus encodes a pseudogene with no associated protein product and that extensive sequencing of human PPCD1 DNA has not revealed mutation or deletion of this gene [16].

Evidence that simple haploinsufficiency of *Csrp2bp* does not cause mouse PPCD1 is the observation that our *Csrp2bp* ^*tm1a*^*/+* and *Csrp2bp*^*Ex*^*/+* mice do not present the mouse PPCD1 phenotype characterized by corneal endothelial cell epithelialization and proliferation and early enlargement of the anterior chamber. An enlarged anterior chamber has not been reported by the International Mouse Phenotyping Consortium (http://www.mousephenotype.org) in *Csrp2bp*^tm1a^ heterozygous or homozygous mice. No eye abnormalities were reported in a separate *Csrp2bp*-haploinsufficient mouse line [34]. However, phenotyping of *Csrp2bp* ^*tm1a(KOMP)Wtsi*^ homozygotes by the International Mouse Phenotyping Consortium has identified a number of eye abnormalities, including corneal opacity, in a fraction of homozygotes. These abnormalities are distinct from those observed in mouse PPCD1, but are consistent with a role for *Csrp2bp* in corneal development. No eye abnormalities have been reported for *Dzank1* null animals by the International Mouse Phenotyping Consortium. Mice haploinsufficient for both *Csrp2bp* and *Dzank1* also do not exhibit PPCD1. Although these double haploinsufficient results must be confirmed in animals lacking the β-Gal-*neo* cassette and crossed fully onto the D2 background, evidence to date suggests that haploinsufficiency of *Csrp2bp*, alone or in concert with *Dzank1*, does not produce the corneal phenotypes seen in the PPCD1 mouse.

The inability of *Csrp2bp* or *Csrp2bp*/*Dzank1* combined haploinsufficiency to produce the mouse PPCD1 phenotype suggests a dominant effect of either the truncated gene product or a novel fusion gene product. In support of this potential mechanism, we have identified a total of 12 alternative transcripts produced as a result of the *Ppcd1* gene rearrangement, one or more of which may act in a dominant manner to deregulate signalling pathways in corneal endothelial cells or precursors. Of particular interest are *Csrp2bp* transcripts C1 and C2, which are overexpressed in PPCD1 eyes.

While this manuscript was under review, Davidson *et al*. reported linkage of mutations in the promoter region of *Ovol2* to human PPCD1 and proposed that human PPCD1 was caused by overexpression of *Ovol2 [19]*. Fig 8 provides evidence of upregulation of *Ovol2* in 14-day old, but not 12-day old, PPCD1 mouse eyes. The *Ovol2* gene is located adjacent to *Csrp2bp*, approximately 37 Kbp 5’ of *Csrp2bp* Exon 1 and 66 Kbp from the 5’-breakpoint of the mouse PPCD1 inversion. Transcription occurs in the reverse orientation. Chromosomal rearrangements are known to perturb expression of neighboring genes [35, 36]; the relatively close proximity and head-to-head orientation of *Ovol2* and *Csrp2bp* are consistent with a direct effect of the PPCD1 chromosomal rearrangement on *Ovol2* transcription. Alternatively, *Csrp2bp* and/or *Dzank1* mutant transcripts may influence *Ovol2* expression and/or function. Further studies on the localization and time course of expression of these three genes are underway to investigate their respective contributions to PPCD1 pathogenesis.

Although *Csrp2bp* haploinsufficiency does not produce the ocular PPCD1 phenotype, our results are consistent with the conclusion that PPCD1 embryonic lethality is due to homozygous disruption of *Csrp2bp*. Wildtype *Dzank1*, *Ovol2*, and *Znf133* alleles are completely unable to rescue embryonic lethality, while introduction of the wildtype *Csrp2bp* does rescue *Ppcd1*-associated embryonic lethality. Rescue is incomplete, which may be due to (1) the presence of *neo* in the *Dzank1*^*Tm1a*^ allele that interferes with *Csrp2bp* expression, (2) the ability of wildtype *Dzank1* to partially compensate for loss of *Csrp2bp* function, or (3) the inability of wildtype *Csrp2bp* to completely compensate for effects of the mutant alternative transcripts. Partial compensation by *Dzank1* and inability of wildtype *Csrp2bp* to compensate for the *Ppcd1* mutation are both consistent with the observation of complete embryonic lethality in *Ppcd1* homozygotes [20] versus partial embryonic lethality of the *Csrp2bp* knockout mouse.

The *Ppcd1* gene rearrangement is expected to alter the functional properties of both the CSRP2BP and DZANK1 proteins, while human PPCD1 *OVOL2* mutations are associated with overexpression rather than a protein structural change. CSRP2BP is a component of the “Ada two A containing” (ATAC) histone acetyltransferase complex, components of which include p300 and P/CAF, transcriptional coactivators involved in *Zeb1* signaling [34, 37–42]. Deletion of *Csrp2bp* Exons 8–11 removes the catalytic histone acetyltransferase domain while leaving intact the amino terminus of the protein, which has the potential to disrupt ATAC function in a dominant negative manner. Although the function of *Dzank1* is unknown, its structure also suggests that it may act as a transcription factor. The PPCD1 chromosomal rearrangement deletes carboxy terminal ankyrin repeats capable of mediating of protein-protein interactions, while leaving the double zinc ribbon DNA-binding domain intact. Finally, overexpression of a wildtype OVOL2 protein may cause PPCD1 via increased repression of *Zeb1* transcription [19, 43].

In conclusion, we have fully characterized the chromosomal rearrangement that causes the PPCD1 phenotype and provide further evidence that disruption of *Csrp2bp*, *Dzank1*, and/or *Ovol2*, singly or in concert, is responsible for the corneal endothelial metaplasia. A number of factors favor *Csrp2bp* disruption as causative for mouse PPCD1. These include *Csrp2bp* gene expression in the corneal endothelium, upregulation of *Csrp2bp* alternative transcripts in affected eyes, inability of the *Csrp2bp* null allele to complement *Ppcd1*-mediated embryonic lethality, and the presence of corneal abnormalities in *Csrp2bp* null animals. The reported associations of CSRP2BP and OVOL2 with *Zeb1* signaling [34, 40–43], known to be disrupted in human PPCD [12, 13], support a role for either or both of these proteins in PPCD1.

## Supporting Information

S1 Fig PCR of distal (3’) breakpoint. Genotypes are indicated at the top and sizes are indicated at the right. +/+denotes wildtype; +/- denotes PPCD1.
(TIF)

S2 Fig Alignment of PPCD1 sequence spanning the distal (3’) breakpoint with GRCm38. The sequence begins in *Dzank1* Intron 19 and spans the breakpoint. The top sequence is that of BAC PL2195, corresponding to the PPCD1 mutant allele. The lower sequence is GRCm38. The PB1D9 SINE element is indicated in bold.
(DOCX)

S3 Fig PCR of proximal (5’) breakpoint. Genotypes are indicated at the top and sizes are indicated at the left. +/+denotes wildtype; +/- denotes PPCD1.
(TIF)

S4 Fig PCR amplification of sequences spanning the proximal breakpoint. Genotypes are indicated at the top and sizes (bp) at the left. +/+denotes wildtype; +/- denotes PPCD1. Primers used were: A, CRIT28854/Sstr7; B, OL8139/CRIT28854; C, OL8140/CRIT28854; D, OL8141/CRIT28854; E, Sstr7/OL8128; F, OL8139/OL8128; G, OL8140/OL8128; H, OL8141/OL8128; I, Sstr7/OL8129; J, OL8139/OL8129; K, OL8140/OL8129; L, OL8141/8129; M, Sstr7OL/8130; N, OL8139/OL8130; O, OL8140/OL8130.
(TIF)

S5 Fig Alignment of PPCD1 sequence spanning the proximal (5’) breakpoint with *Csrp2bp* Intron 7 and GRCm38. The top sequence is that of PPCD1, derived from sequencing of PCR amplicons shown in S1D Fig. The lower sequence is that of *Csrp2bp* Intron 7 sequence (labelled Int_7), obtained from the wildtype BAC clone, PL2173 (shown in S1E Fig), or GRCm38. The B2_Mm1a SINE element, present in D2 but not B6, is shown in bold. Positions of oligonucleotides used for determination of the sequence spanning the breakpoint are indicated by arrows.
(DOCX)

S6 Fig Sequence of D2 *Csrp2bp* Intron 7. Sequence derived from the wildtype BAC clone, PL2173.
(DOCX)

S1 Table Primers used in this study. Primers are arranged in pairs. Amplicon sizes, if applicable, and the usage of each primer or primer pair are indicated.
(XLSX)
