# Supplementary figures and images for: Association of a Chromosomal Rearrangement Event with Mouse Posterior Polymorphous Corneal Dystrophy and Alterations in Csrp2bp, Dzank1, and Ovol2 Gene Expression
Source: PLoS One. 2016 Jun 16;11(6):e0157577. doi: 10.1371/journal.pone.0157577 (PMC4910986; doi:10.1371/journal.pone.0157577)

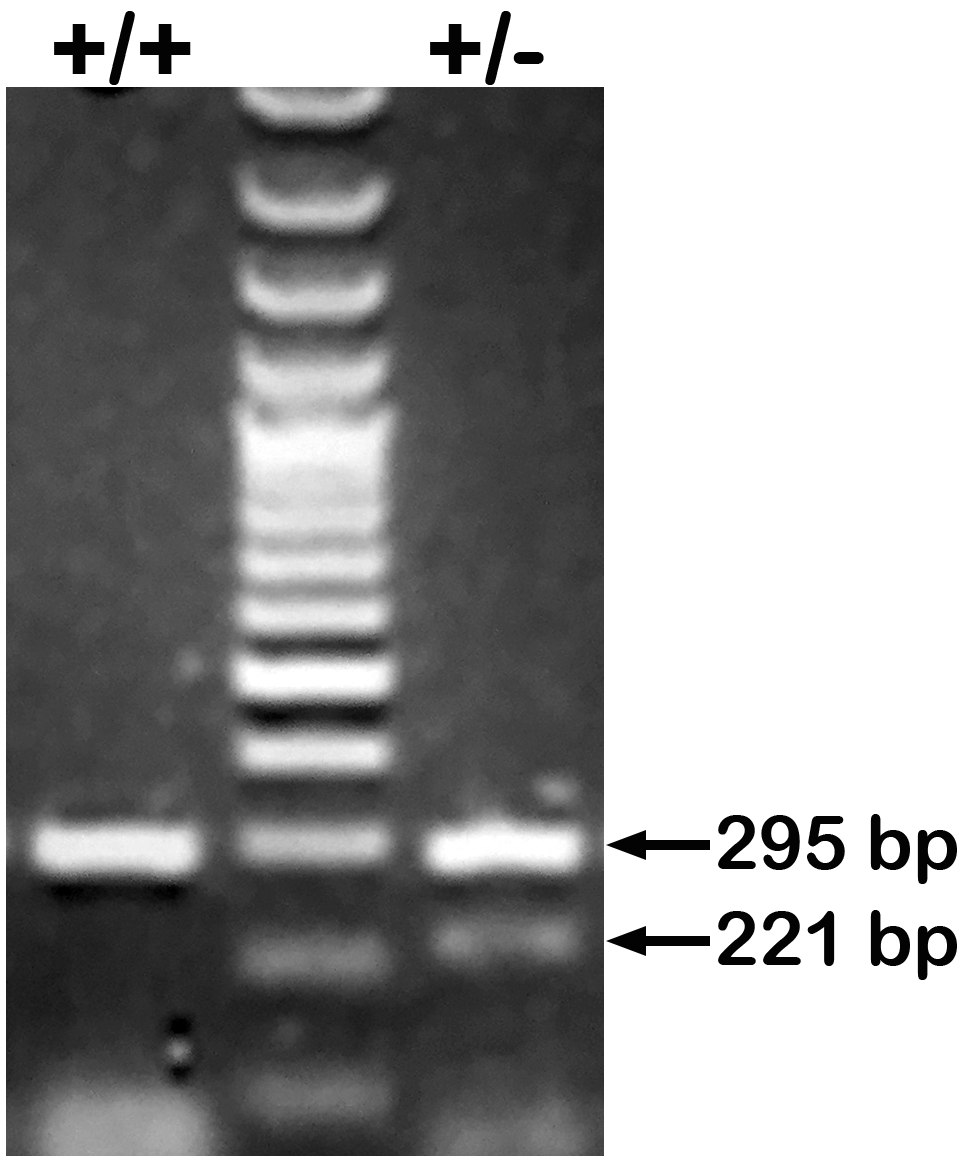

Supplement: S1 Fig — Genotypes are indicated at the top and sizes are indicated at the right. +/+denotes wildtype; +/- denotes PPCD1. (TIF) [file pone.0157577.s001.tif]

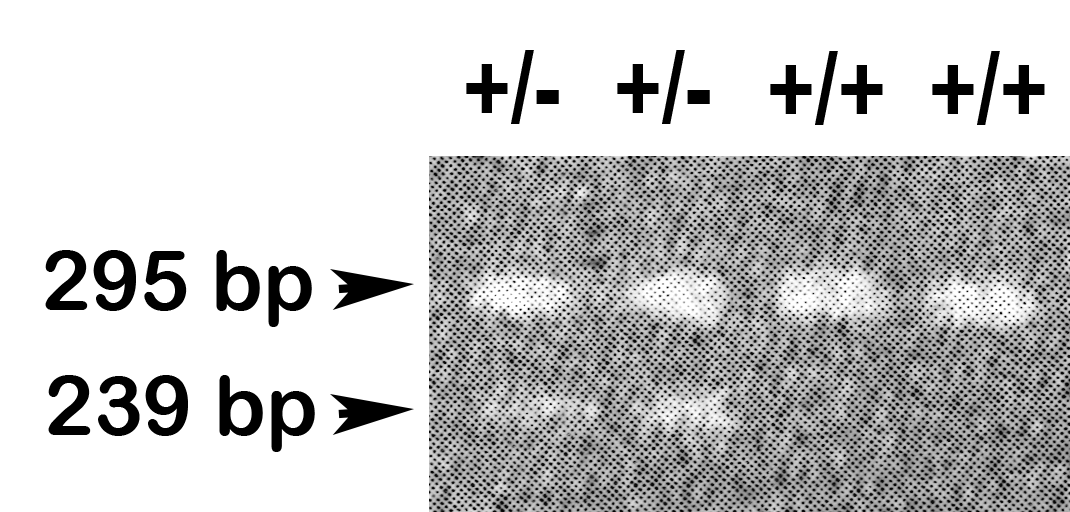

Supplement: S3 Fig — Genotypes are indicated at the top and sizes are indicated at the left. +/+denotes wildtype; +/- denotes PPCD1. (TIF) [file pone.0157577.s003.tif]

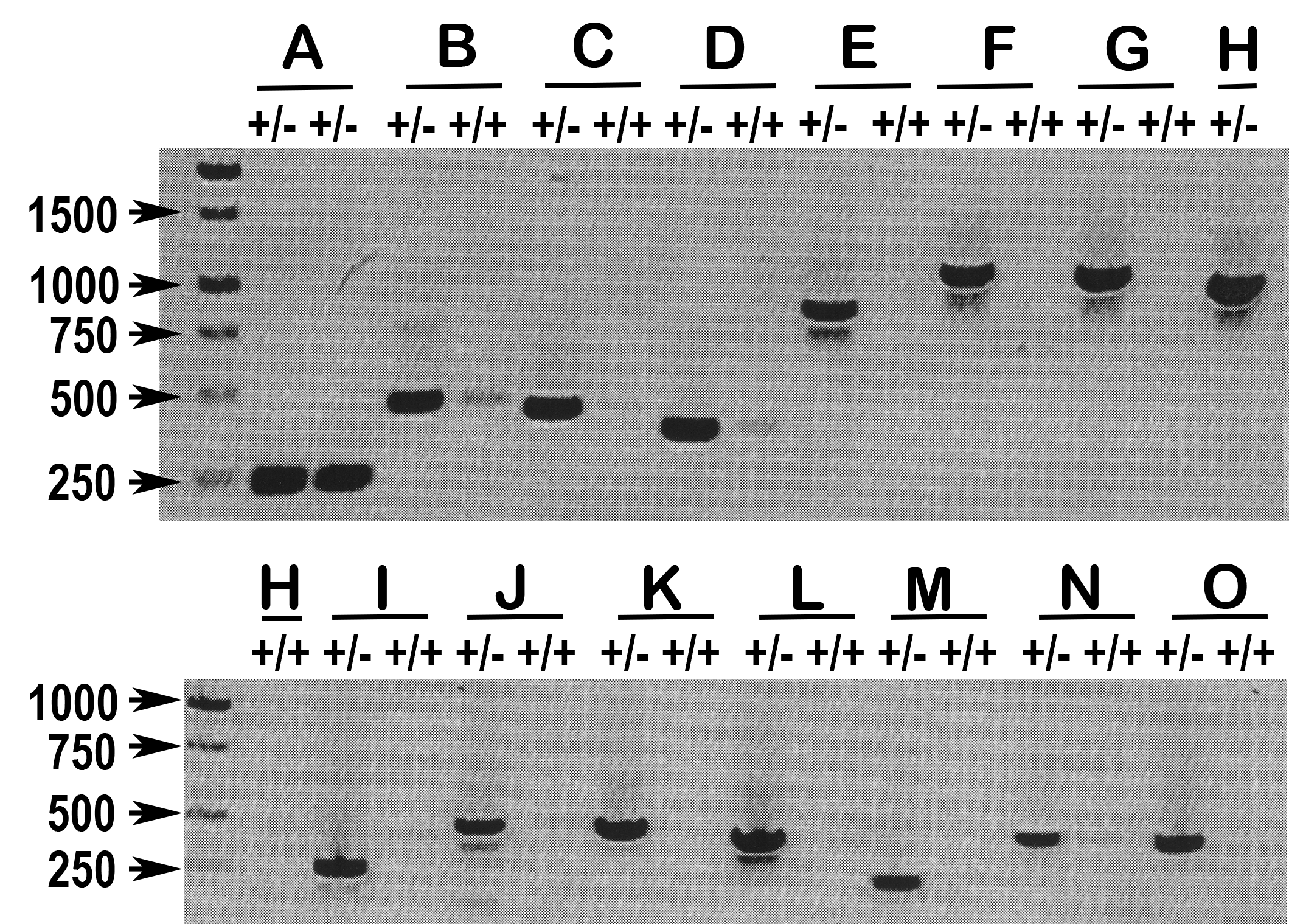

Supplement: S4 Fig — Genotypes are indicated at the top and sizes (bp) at the left. +/+denotes wildtype; +/- denotes PPCD1. Primers used were: A, CRIT28854/Sstr7; B, OL8139/CRIT28854; C, OL8140/CRIT28854; D, OL8141/CRIT28854; E, Sstr7/OL8128; F, OL8139/OL8128; G, OL8140/OL8128; H, OL8141/OL8128; I, Sstr7/OL8129; J, OL8139/OL8129; K, OL8140/OL8129; L, OL8141/8129; M, Sstr7OL/8130; N, OL8139/OL8130; O, OL8140/OL8130. (TIF) [file pone.0157577.s004.tif]
