# Supplementary material for: Association of a Chromosomal Rearrangement Event with Mouse Posterior Polymorphous Corneal Dystrophy and Alterations in Csrp2bp, Dzank1, and Ovol2 Gene Expression
Source: PLoS One. 2016 Jun 16;11(6):e0157577. doi: 10.1371/journal.pone.0157577 (PMC4910986; doi:10.1371/journal.pone.0157577)
Supplement: S2 Fig — The sequence begins in Dzank1 Intron 19 and spans the breakpoint. The top sequence is that of BAC PL2195, corresponding to the PPCD1 mutant allele. The lower sequence is GRCm38. The PB1D9 SINE element is indicated in bold. (DOCX) [file pone.0157577.s002.docx]

S2 Fig. Alignment of sequences spanning the 3’-breakpoint with GRCm38. The PPCD1 sequence is taken from the BAC clone spanning the breakpoint.

**PPCD1** 1 GCACTTATAAAATATGTCACTATGTTTGT-GGTACACACCTGCAGTTTTAGCACTTAGGA 59

||||||||||||||||||||||||| ||| |||||||||||||||| |||||||||||||

**GRCm38** 144477856 GCACTTATAAAATATGTCACTATGTCTGTTGGTACACACCTGCAGTCTTAGCACTTAGGA 144477797

**PPCD1** 60 AGCCGAAACAGGAGGACCATGAATTTGAGGCTAGACAGGTCTATGATAACAAGAGCCTGT 119

|||||||||||||||||||||||||||||||||||||||||||||||||||||||||||

**GRCm38** 144477796 GGCCGAAACAGGAGGACCATGAATTTGAGGCTAGACAGGTCTATGATAACAAGAGCCTGT 144477737

**PPCD1** 120 CTTAAAACACAGGAACTATATGTTTTTTTCTGAGAGCATTTTTGTATTTTCCATAAGACG 179

||||||||||||||||||||||||||||||||||||||||||| ||||||||||||||||

**GRCm38** 144477736 CTTAAAACACAGGAACTATATGTTTTTTTCTGAGAGCATTTTTATATTTTCCATAAGACG 144477677

**PPCD1** 180 CATATATGAGAAGATACTGCTCCCTTTCAGTTTTATTTTATATTTGATATATATTATAAA 239

||||||||||||||||||||||||||||||||||||||||||||||||||||||||||||

**GRCm38** 144477676 CATATATGAGAAGATACTGCTCCCTTTCAGTTTTATTTTATATTTGATATATATTATAAA 144477617

**PPCD1** 240 GCTTTGTTGACTTTCTAAAGTTTCCTGAAGGTTTCTATTCACTATGCTACCACCCCCTTC 299

|||||||||||||||||||||||||||||||||||||||||| ||||||||||

**GRCm38** 144477616 GCTTTGTTGACTTTCTAAAGTTTCCTGAAGGTTTCTATTCAC--------CACCCCCTTC 144477565

**PPCD1** 300 GTGTTCCACACCTTTGCCTCATTGTCTTGATCATGAAATGATGCTATTTGTTTTGGTTTT 359

|||||||||||||||||||||||||||||||||||||||||||||||||||||||||||

**GRCm38** 144477564 GTGTTCCACACCTTTGCCTCATTGTCTTGATCATGAAATGATGCTATTTGTTTTGGTTTG 144477505

**PPCD1** 360 GTTTTTTGCGAGGCGGGGGGCGGAGGGGGATAATAAGGT**CTCACTGTATAGCCCTGGATG** 419

||||||||||||||||||||||||||||||||||||||||||||||||||||||||||||

**GRCm38** 144477504 GTTTTTTGCGAGGCGGGGGGCGGAGGGGGATAATAAGGTCTCACTGTATAGCCCTGGATG 144477445

**PPCD1** 420 **GCCTTGAATTCACAAAGATTCACCTGCCTCTGCATCCTGAGTACTG**CAATCCAAGGTGTG 479

||||||||||||||||||||||||||||||||||||||||||||||||||||||||||||

**GRCm38** 144477444 GCCTTGAATTCACAAAGATTCACCTGCCTCTGCATCCTGAGTACTGCAATCCAAGGTGTG 144477385

**PPCD1** 480 TACTACTGTGCCCAGAGATGCTATTTGCTCTATAATATACTTTATATAATCCAAACCAAC 539

||||||||||||||||||||||||||||||||||||||||||||||||||||||||||||

**GRCm38** 144477384 TACTACTGTGCCCAGAGATGCTATTTGCTCTATAATATACTTTATATAATCCAAACCAAC 144477325

**PPCD1** 540 TTTTCATAGCACCTAAGCATGATGCCTTATGCCTGATACACAAGTCATTTTTTGAATGAT 599

||||||||||||||||||||||||||||||||||||||||||||||||||||||||||||

**GRCm38** 144477324 TTTTCATAGCACCTAAGCATGATGCCTTATGCCTGATACACAAGTCATTTTTTGAATGAT 144477265

**PPCD1** 600 TCTTCAGTGAAATGAGTTCTAGATTCACGAACAGTCCAACTAAGTCTAAGACTAAACTGT 659

||||||||||||||||||||||||||||||||||||||||||||||||||||||||||||

**GRCm38** 144477264 TCTTCAGTGAAATGAGTTCTAGATTCACGAACAGTCCAACTAAGTCTAAGACTAAACTGT 144477205

**PPCD1** 660 ACATAGCACAAGTTGC--T---A-TG-----TTCCTTAGTGTCCTTACGTACTTCATCAG 708

|||||||||||||||| | | || |||||||||||||||||||||||||||||

**GRCm38** 144477204 ACATAGCACAAGTTGCCATTTTAATGTGCGCTTCCTTAGTGTCCTTACGTACTTCATCAG 144477145

**PPCD1** 709 CACTCCACAGTGACACAGATGCAGGTGTGAGTCCAGCTGACTAGTGAGACAGTACCACTG 768

||||||||||||||||||||||||||||||||||| ||||||||||||||||||| ||||

**GRCm38** 144477144 CACTCCACAGTGACACAGATGCAGGTGTGAGTCCATCTGACTAGTGAGACAGTACTACTG 144477085

**PPCD1** 769 CCTCTCTGCTAAGCCCCAGGACTGAGA 795

|| ||||||||||||||||||||||||

**GRCm38** 144477084 CCGCTCTGCTAAGCCCCAGGACTGAGA 144477058

**PPCD1** 794 GATATGAGACGCCTCATTTTTAAGGAGCTCATAACCCAGGCAGGAAGGAAAATCATCACC 853

||||||||||||||||||||||||||||||||||||||||||||||||||||||||||||

**GRCm38** 148326553 GATATGAGACGCCTCATTTTTAAGGAGCTCATAACCCAGGCAGGAAGGAAAATCATCACC 148326612

**PPCD1** 854 ACATGAAAAGGCCACCACCACACACAGTGTGAACATCTGTACTTGATTGAGGGGAGAAAA 913

||||||||||||||||||||||||||||||||||||||||||||||||||||||||||||

**GRCm38** 148326613 ACATGAAAAGGCCACCACCACACACAGTGTGAACATCTGTACTTGATTGAGGGGAGAAAA 148326672

**PPCD1** 914 CTGCTTTCCCCCCAGAGTGGGACTCTGCTGGAAATGCTATAAACATCTTCAGTGAATCGA 973

||||||||||||||||||||||||||||||||||||||||||||||||||||||||||||

**GRCm38** 148326673 CTGCTTTCCCCCCAGAGTGGGACTCTGCTGGAAATGCTATAAACATCTTCAGTGAATCGA 148326732

**PPCD1** 974 TTCCCACCACTGCAAGGACTGTTGTTTTCCCTTAACCCACAGGAAATTCAATAAATAAAA 1033

||||||||||||||||||||||||||||||||||||||||||||||||||||||||||||

**GRCm38** 148326733 TTCCCACCACTGCAAGGACTGTTGTTTTCCCTTAACCCACAGGAAATTCAATAAATAAAA 148326792

**PPCD1** 1034 GAGACTTTAACTGGGATCAGGAAAAAGACAGGGTTTTTTTGTGTCAGTTCAGGGAGAACG 1093

||||||||||||||||||||||||||||||||||||||||||||||||||||||||||||

**GRCm38** 148326793 GAGACTTTAACTGGGATCAGGAAAAAGACAGGGTTTTTTTGTGTCAGTTCAGGGAGAACG 148326852

**PPCD1** 1094 GGATGGCACAGAGCTGACCTC 1114

|||||||||||||||||||||

**GRCm38** 148326853 GGATGGCACAGAGCTGACCTC 148326873
