# Supplementary material for: Association of a Chromosomal Rearrangement Event with Mouse Posterior Polymorphous Corneal Dystrophy and Alterations in Csrp2bp, Dzank1, and Ovol2 Gene Expression
Source: PLoS One. 2016 Jun 16;11(6):e0157577. doi: 10.1371/journal.pone.0157577 (PMC4910986; doi:10.1371/journal.pone.0157577)
Supplement: S5 Fig — The top sequence is that of PPCD1, derived from sequencing of PCR amplicons shown in S1D Fig. The lower sequence is that of Csrp2bp Intron 7 sequence (labelled Int_7), obtained from the wildtype BAC clone, PL2173 (shown in S1E Fig), or GRCm38. The B2_Mm1a SINE element, present in D2 but not B6, is shown in bold. Positions of oligonucleotides used for determination of the sequence spanning the breakpoint are indicated by arrows. (DOCX) [file pone.0157577.s005.docx]

S5 Fig. Alignment of PPCD1 sequence spanning 5’-breakpoint with Csrp2bp Intron 7 (labelled Int_7) and GRCm38. The Csrp2bp Intron 7 sequence is taken from the sequence of the wildtype BAC clone and is shown in Figure S1C. The LINE insertion present in DBA/2J but not C57BL6J is shown in bold. Positions of oligonucleotides used for determination of the sequence spanning the breakpoint are shown.

OL8128

**PPCD1** 1 GAGGAGGGTTGTAGCCTCGTGAGCCCTTCCCCACCCTCTGGGGGRTAGTTAGTGAGCCCA 60

|||||||||||||||||||||||||||||||||||||||||||| |||||||||||||||

**Int 7** 3037 GAGGAGGGTTGTAGCCTCGTGAGCCCTTCCCCACCCTCTGGGGGATAGTTAGTGAGCCCA 3096

OL8129

**PPCD1** 61 ATCTTGTTTAGGTCTCCTTCAGGAAATCATAGCTGCTGATGGGTCTAGAGGGCACCGGCC 120

||||||||||||||||||||||||||||||||||||||||||||||||||||||||||||

**Int 7** 3097 ATCTTGTTTAGGTCTCCTTCAGGAAATCATAGCTGCTGATGGGTCTAGAGGGCACCGGCC 3156

**PPCD1** 121 ATGGCGTGCCTGAAGGGTGGCATTCCACGGCACAGGTGTTATCTCAGCTTCCTCCATCTG 180

||||||||||||||||||||||||||||||||||||||||||||||||||||||||||||

**Int 7** 3157 ATGGCGTGCCTGAAGGGTGGCATTCCACGGCACAGGTGTTATCTCAGCTTCCTCCATCTG 3216

OL8130

**PPCD1** 181 AGGTGTGGTGTCCCACCCTAGTCAGAGCTGTAACTGCTGCACGCGTGTTTTATGTCCCCT 240

||||||||||||||||||||||||||||||||||||||||||||||||||||||||||||

**Int 7** 3217 AGGTGTGGTGTCCCACCCTAGTCAGAGCTGTAACTGCTGCACGCGTGTTTTATGTCCCCT 3276

OL8131

**PPCD1** 241 TTCCTTCTGCTGTTCCTGTCATCCTTTCCTGGTTTCCCACAACTCTGGGACATTCGATTC 300

||||||||||||||||||||||||||||||||||||||||||||||||||||||||||||

**Int 7** 3277 TTCCTTCTGCTGTTCCTGTCATCCTTTCCTGGTTTCCCACAACTCTGGGACATTCGATTC 3336

**PPCD1** 301 CACTCATTTATAGTCTTTTTTCTCTGCCTTTGGTTTGGGAAGGTTTTATGCAGCTGCTCA 360

||||||||||||||||||||||||||||||||||||||||||||||||||||||||||||

**Int 7** 3337 CACTCATTTATAGTCTTTTTTCTCTGCCTTTGGTTTGGGAAGGTTTTATGCAGCTGCTCA 3396

OL8132

**PPCD1** 361 AGCTCAGAACGACTCTTCTAAACTATGCATAGAAGGTTATTGATTAGTCCACTGTCAAAA 420

||||||||||||||||||||||||||||||||||||||||||||||||||||||||||||

**Int 7** 3397 AGCTCAGAACGACTCTTCTAAACTATGCATAGAAGGTTATTGATTAGTCCACTGTCAAAA 3456

**PPCD1** 421 CGGCTTAGTTTCAGTTGCTGtttttttt**ttttttttttAAATTTATTTATTTATTATATG** 480

||||||||||||||||||||||||||||||||||||||||||||||||||||||||||||

**Int 7** 3457 CGGCTTAGTTTCAGTTGCTGTTTTTTTT**TTTTTTTTTTAAATTTATTTATTTATTATATG** 3516

**PPCD1** 481 **TAAGTACACTGTAGCTGTCCTCAGACACTCCAGTCAGATCTTGTTACAGATGGTTGTGAG** 540

||||||||||||||||||||||||||||||||||||||||||||||||||||||||||||

**Int 7** 3517 **TAAGTACACTGTAGCTGTCCTCAGACACTCCAGTCAGATCTTGTTACAGATGGTTGTGAG** 3576

**PPCD1** 541 **CCACCATGTGGTTGCTGGGATTTGAACTCCTGACCTTCGGAAGAGCAGTCGGGTGCTCTT** 600

||||||||||||||||||||||||||||||||||||||||||||||||||||||||||||

**Int 7** 3577 **CCACCATGTGGTTGCTGGGATTTGAACTCCTGACCTTCGGAAGAGCAGTCGGGTGCTCTT** 3636

CRIT28854F454

**PPCD1** 601 **ACCCACTGAGCCATCTCACCAGCCCTC**AGTTGCTGTTTTTGCCCTCTGCATTAATCTACT 660

||||||||||||||||||||||||||||||||||||||||||||||||||||||||||||

**Int 7** 3637 **ACCCACTGAGCCATCTCACCAGCCCTC**AGTTGCTGTTTTTGCCCTCTGCATTAATCTACT 3696

**PPCD1** 661 TCTTTCTTAGGACTTCCAGCCCTTTCTAAACTGCCATCTGCTCTTGCATGCT 712

||||||||||||||||||||||||||||||||||||||||||||||||||||

**Int 7** 3697 TCTTTCTTAGGACTTCCAGCCCTTTCTAAACTGCCATCTGCTCTTGCATGCT 3748

**PPCD1** 713 TCACTTGAGTTGCTGCTTCTCTATCCGGGAAGTGGGGACACAAGCTCTCTGCATTCGCAT 772

||||||||||||||||||||||||||||||||||||||||||||||||||||||||||||

**GRCm38** 148326011 TCACTTGAGTTGCTGCTTCTCTATCCGGGAAGTGGGGACACAAGCTCTCTGCATTCGCAT 148325952

**PPCD1** 773 CCAGAGAGATACACAAGAGGAAACAGATGTAGAAAATAAGACAGTGTGGTATTGATGAGT 832

||||||||||||||||||||||||||||||||||||||||||||||||||||||||||||

**GRCm38** 148325951 CCAGAGAGATACACAAGAGGAAACAGATGTAGAAAATAAGACAGTGTGGTATTGATGAGT 148325892

Sstr7

**PPCD1** 833 ACATTCTGGTTTTCCCAAGGGCAAGAAACTCATGTCTTTCCATCTCTATGTGCAAACCAA 892

||||||||||||||||||||||||||||||||||||||||||||||||||||||||||||

**GRCm38** 148325891 ACATTCTGGTTTTCCCAAGGGCAAGAAACTCATGTCTTTCCATCTCTATGTGCAAACCAA 148325832

**PPCD1** 893 GACTTGTATCATTTTCTAATTATCTTTTAAATAAAATATAAAGTGGAGATTTGGCTTTGT 952

||||||||||||||||||||||||||||||||||||||||||||||||||||||||||||

**GRCm38** 148325831 GACTTGTATCATTTTCTAATTATCTTTTAAATAAAATATAAAGTGGAGATTTGGCTTTGT 148325772

OL8141

**PPCD1** 953 AGTGGAACACTTAAAAGCRAGGTCCTGGGTGTGATTACCAGCATTGAGGGAGGAAAGTGA 1012

|||||||||||||||||| |||||||||||||||||||||||||||||||||||||||||

**GRCm38** 148325771 AGTGGAACACTTAAAAGCAAGGTCCTGGGTGTGATTACCAGCATTGAGGGAGGAAAGTGA 148325712

OL8139

OL8140

**PPCD1** 1013 CATTTTAAGAGAGAGAGAAAATGAATGTGATGCAAAGAGCTGACCTCAGGAGGATTGGTA 1072

||||||||||||||||||||||||||||||||||||||||||||||||||||||||||||

**GRCm38** 148325711 CATTTTAAGAGAGAGAGAAAATGAATGTGATGCAAAGAGCTGACCTCAGGAGGATTGGTA 148325652

**PPCD1** 1073 CCCTGA 1078

||||||

**GRCm38** 148325651 CCCTGA 148325646
