# Supplementary material for: Association of a Chromosomal Rearrangement Event with Mouse Posterior Polymorphous Corneal Dystrophy and Alterations in Csrp2bp, Dzank1, and Ovol2 Gene Expression
Source: PLoS One. 2016 Jun 16;11(6):e0157577. doi: 10.1371/journal.pone.0157577 (PMC4910986; doi:10.1371/journal.pone.0157577)
Supplement: S6 Fig — Sequence derived from the wildtype BAC clone, PL2173. (DOCX) [file pone.0157577.s006.docx]

S6 Fig. Sequence of DBA/2J Csrp2bp Intron 7. The LINE insertion present in DBA/2J but not C57BL6J is shown in bold.

GTGATGTTACTGTACTGTGACATTTGTCATTGTGAGCAATCTGGGGAGAAGAAAGCAAGCAAAACTTCTCTGCTTCTCTTCCAAAACTGTTCATCATTTAGTGCCCCTTCCTGTATCTGAGTTTCTTTCTCACTAGGCATTCAGGGAACAGCCTAGCCATGTCTCGCCTCTCTCCCTTGAGGGTATCTGTCTGTGGTCTGTTTCATGGTTGTGATTGACAGGTTTGTTGAGGACATGTATAGCCTAGACCCTGTCGAGCTGTGGGGGTAAGCATCTGTGTCCTTAGGCTTCATCAGGAAGAAATGCGGGGTCAGCATTACTCACAGGGTGAGTTCTGCCAATGCTCTGACAACATTCATTCCCTCGGGGGCTATGGGCTGCCCCACCAGCAGCGGGTTCTGTTTCCTCTGTTCTGCCCTGTTGTTTTGAATTGGCATTCCTCATGTGTAAAGGTGCTGATGTGAGTACTGTCGGCTCTCTACGCTAGCCACATAGTCTGCAAGGGACCGAGACCTGCCTGTGCTGGACACAGTGGTAGTAGTCAGTGTTCGCAGCTGTGCCCACCCCAGTGAGCTCGTTTACTAAAACGGTCTCAGGCTGGGCTGGCCTGCAGGGATCCATCACCAGGACTGCTCTAGGTCCTGACTTAAGATGTGGTTTTCTGTACTGTTAAGTGTTGTGTTGATGGCCCCACCCTCACTGCCTTTCCTTGCTCTCTTTCCTTTTGTGTTGTTTATATAAATGTTAGTGGACTCTGATTTTTTCTTTATTTCTCCCTGTACTAACTTCTAGCTGGATTCTTCTTTCTTTCTTTCTTGATTTACTTATTATTATAAATAAGTACACTGTTGCTATCTTCAGACACTTCAGAAGAAGGCGTCAGATCTCATTACGAATGGTTGTGAGCCACCATGTGGTTGCTGGGATTTGAACTCAGGACTTTCAGAAGAGCACTCAGTGCTCTTACCTGCTGAGCCATCTCGCCAGCCCTGGACTGCTTTCTCTAGCAGTTCTTTACAACATGGTTTCTTTTAACATACATACATACATACATACATACATTTATGTATTGTGTGCATGTGCATACATACCACAGCACATATGTGGGGTCAGAAGACAGCTTGCTGTAGTTGGTTCTCTTCTAGCACATGGGTTTTTAGTGTTAGAGTCGGGTCTCAGGCTTTACCTAATGAGCCATCTCACCATCCCTAAATATGAACTTTTTGAGGTATATTTTGAAAACCTGTGTGCCTAGAATATCTTTATTTCACTTAAAAAAAAAAAGATGAATTTATTTATGTGAGTACACTGTTGCTCTCTGCAGACACATCAGAAGAGGGCATCAGATTCCATTACATATGGTTGTGAGCACCATGTGGTTGTTGGGAATTGAACTCAGGACCTCTGGAAGAGCAGTCAGTGCTCTTAACCTCTGAGCCATCTCTCTGGCCCTTTATTTCACTCTTTTTTTTTTTTTTTTTTTTTTGGTTTTTTGACACAGGGTTTCTCTGTGTAGTCTTGGCTGTCCTGGAACTCACTCTGTAGACCAGGCTGGCCTCGAACTTAGAAATCCGCCTGCCTCTGCCTCCGAGTGCTGGGATTAAAGGTGTGCACCACCACGCCCGGCTCCTTTATTTCACTCTTACATCAACAATACCTTACAAAGTTTTAGTTTTGTTTTCTCCTTTGACATTTTGAAGTTGTAACTTCATTATCATCTTATGTACTGGTTGCTCCTGAGAGCCCAGTTTCTTTTGTTATTATTTGAGACAGGGTCTCACTGTATAGCGCTGAACTGAAACTCAGGTTTGCCTCTCTTCTGAGTGTTGGAATTAACAGTGTGAGCTACCTTGTCTTGCCATGAGAACCCCATTTCCAATGTTAATTAATTAATTAATTAATTAATTTTTGGTGTTTTTTTTTTTTTTTAATTCTCCTGTCTTCTGGAAGCTTCCATATTTTCTGTTTCTGATCTTCAGTGTATTTTCACTGCTTTTTCTGTCTTTTGTATCTCACAACTCTACTCTTGGGTTAGCAGTTGTTGTGTGAACCTTTCCTGGGGAGAAACAGATGTCTCTTTGCCCCAGTTCTGCCACCAGCAGTAGACCAAAACAGCAGTTCCTGTCACGTCCATGAGTTTAATTAGGGTCCCTGCTTTAGTTTGCATACCTATTTCTGTGAGAAAATACCCTGACAAAAGTGACTTAAGGGAGAGAGGGTTTCTCTTGACTCATGGCTCCAAGTACAGTCCACTATGGTGAGGAAGTTGGAGCATGGAGAGCTTGAGGGAGCTGGTCAGATGGAATCTACTGTCCCAGCTGGGTAATGGCCCCACCCACAGTGGGCAAGTCTTCCCTCTTTAGTTAATGCCATCCAGATAACCCCCAACAGGCATTCCTAGATGTCAGTTTCCCAGGTGCTCCTAGATTAAGTCTCATTGACAGTTAACACTAGACATCACAATTCCTTGCAGGAATGGAAAGGTTAACTATAGGAGCATGGGCACCTGATGGGCAGCCATACCACTGGAGGGGAAAACCTCTGCATTAACTGTTCATAAATCCTGGGCACAGGGCTGGAGAGATGGCTCAGTGGCTAAGAGCACTGGCTGATGCTCTTCCAGATAGACTGAGTTGAATTCCCAGCAACCTCATGATGGCTCACAACTATCTGTAATACGATCTGATGCCCTCTTCAGGCATGTAGGTGTACATACAGATAGAACACTCAGGCACATAATAAATGAATACATCTTAAAAACAAAACAAAAAAAGGTATGTGCCACAATAAATATTGGCTGGGCCAATAAATACTTAAAAAAAAGACAATCTTGGGGGGTGGGTATGGGGGACTTTTGGGATAGCATTGGAAATGTAAATGAGGAAAATACCTAATTAAAAAAACATATATATATATACATATATATATGTATATATATATATATATATATATGTATATATATATATAAAAGACAATCTTGGGCAGAGGTGTGACCTCGTGAGCACCCTGTATTAACCATTGACCATCTATAGCTtGGAGgAgGGTTGTAGCCTCGTGAGCCCTTCCCCACCCTCTGGGGGATAGTTAGTGAGCCCAATCTTGTTTAGGTCTCCTTCAGGAAATCATAGCTGCTGATGGGTCTAGAGGGCACCGGCCATGGCGTGCCTGAAGGGTGGCATTCCACGGCACAGGTGTTATCTCAGCTTCCTCCATCTGAGGTGTGGTGTCCCACCCTAGTCAGAGCTGTAACTGCTGCACGCGTGTTTTATGTCCCCTTTCCTTCTGCTGTTCCTGTCATCCTTTCCTGGTTTCCCACAACTCTGGGACATTCGATTCCACTCATTTATAGTCTTTTTTCTCTGCCTTTGGTTTGGGAAGGTTTTATGCAGCTGCTCAAGCTCAGAACGACTCTTCTAAACTATGCATAGAAGGTTATTGATTAGTCCACTGTCAAAACGGCTTAGTTTCAGTTGCTGTTTTTTTT**TTTTTTTTTTAAATTTATTTATTTATTATATGTAAGTACACTGTAGCTGTCCTCAGACACTCCAGTCAGATCTTGTTACAGATGGTTGTGAGCCACCATGTGGTTGCTGGGATTTGAACTCCTGACCTTCGGAAGAGCAGTCGGGTGCTCTTACCCACTGAGCCATCTCACCAGCCCTC**AGTTGCTGTTTTTGCCCTCTGCATTAATCTACTTCTTTCTTAGGACTTCCAGCCCTTTCTAAACTGCCATCTGCTCTTGCATGCTCGCCCCTTAGAGCATGTAACATATCAACTACCGCTGTTAAATGTCTTTCCCAGGAATCTCAGGACCCCTGCACTGAGTGAGTGTGCTTCTGGGGCTTGTTTGTCTCTTCAAGTGGTCTAGCCATGGTGGCTCAGTGTTCCGTGAAGTACGAGGCTCTGTTCCATCACATCCATGCCCCCGTCGCACCCCCATCAGCTCAGGCAGCTGCCCTCCCCACATTCCCAGGCACCCATGCTACTGTTTTATACCTCCAGTGTATATTCCTGGGATATGGAACACTTAGTTCACCACAGTGGTGCCTTTCCTCTGTCTCAGGAAGAGTCCAACAGACACTCAGAGCAGCCCCTGAGAGAGGTGTAATGAGGCCTTTCTAGCCCCTCTGGTGCAGAGCCTGCTCTGTTCTCATTGCATTGCTAGTTCTAGCCAGCTTTTCCTTCTAGGCTTTTCCTCAGCCTGCATTAGTAAAGAAGCACACTTAACGCTGTCACATGTCACTTGACTTTGAAGCTGTTTTTTCTCCACCACTCTGCGATTTGGTAAATTGAGTAGTGCACTATTCTAACTGGGATGCCAATGCTAGGTAGGTTTCTGTCCTCTGCAGCTGACAGTCTCAAACAGGGAAGTGACAGGGTTAGCTCACTAAGCTAAGATGCACTGACTGAGGGCGTGTGTATGTGTGTGTGTGTGTGTGTGTCACGGAAGGACTGCTGTGTTCCTGGGAATTAAAACCAGCCTCTGAGGGAGGTAACCTTGCAAGAAGTGCTGACAAGGAAATGCCAGGAGGAAGAAAACTGGATGGAAGGCCAGACCCAGGCCCTGAGGCATGAGAAAGACTGGTAGCTGTGATGGGAACATCCTGACGACACAGGAGTGTGGTTCTAGGAGGAGAGGCCAGAGTGTGACAGAGTCAGGTCTTGCAAGTTCCCTGTAAGGCACAGTTGAGGACTTTGAAATGTACTCTGTACATACAGTGGGAAACCATATAGGAATTGTGAACCAGGGGGCATGAGTTGATGCTTGCTTTTTAAATAGAGTCTTACTGTGTAGCCCTGACTGGACTAAATCTCACTTTGTAGACCAAGCTGGCCTTGAACTCACAGAGATCTGCATGTCTTTGCCTCCCAAGTGTTGGGATTAAATGTGTGTTGCCACTATGCCTAGTTAGTTTATTTTTAAAAGAAGTGTGTGGCCTCTATGTGGAGACTGGATGGTGTGGGACAAGAATGGGCCAAGAGGAGGCAGTGATGGTGGTCCAGGCTTGGTGGACCGTGTTTTGGAGAAGAGTAGAAGTTGCAGAGGTTAGCGGAACTTTGTCACATCCATGAGATTCTACAGGACTCACATGAGACTGTTAAGGGGGAGAAATTGTTCTGTGGACTGATAGATCTACAAATTATCTGGCATGGCATTGAAAGCCACCCCCAAACTTGGTGACTTGATGTAATGAGTACTAAATATGTCTTGAACTTCTGCCTCTGCCTGGCCTCAGCAGAGTGGCTCTTTTACTTTGTGAGTCATTGGGGTCTTGGTTCCATGGGGCCTGTGGTCATCCACTGGCCTAGTTGGAGCCACCTGGAAGCTACATGCGCATTGTGCATGGAGTGGAAGACTTGGGCTTGAAGCGCCAGGCATTGCTTCTGCCACTCTGATTGGTCAAAGTTCTTTACAGCCATTATACCTGCAAGGGTGGGGGGTGTTGCTAGTGGCTGTGTTTGGAAACTGCTTGCGCAGCTTTGAAGATTGCTTTGAAATTCACTTGAAGCCTTGGAAGAATTTCAAAGCTGGCTTTTGAGGAGTTTGGTGTCCAGGCTAGAGAAGTCTGGAAGCAAATTACAAAGGAAGAAACCTTGCTCTTTTCAACAAGTTATTTTCCAGGCTAAGAGCAAACTGACTCTAAAAGACAACTCGGTTTCTAAGCTTCACCTTGACACTCCCTTTGTGAAGGTCTTTTCATCTTTGTTGGAGTTCTTTGCTATTACAGCCATAATTGCGGTGTTCTGTTGCTACATGACCTAGGGCCTCAGTTAGCAGTCCTCAGAAGAGAGCAGCACACCAGCAGTGTGGCCAGTTCCTCTCTGGAGCTTCCAAACCAAGCATGTGAAACCCAGGAGTTCTGTCCCAACTCCTGCCCAGCCAAGGCCACATCTATGCCTCGTCTCTGCTGGGTGGTGCCTCCTTCCTGTGCTGACAAGTCTAGGGCTAGTAATCAATATCACTCCCGTGTTTGCCATGTTCACTTGCTGTAAAGAGTACACAGTCGCCGGGCATGGTGGCGCACACCTTTAGTCCCAGCACTTGGGAGGCAGAGGCAGGCGGATTTCTGAGTTCGAGGCCAGCCTGGTCTACAAAGTGAGCTCCAGGACAGCCAGGGCTATACAGAGAAACCCTGTCTCGAAAAAACAAGACAAAACAAAAAAAAGAGTACACAGTCAAGGGGTGGGGCATTGGGCCTGTTGGTTGTTAACCATAATATAACAACATAAGGTTGCATAATAAATAATAAATAGCCCACCTATGTTAGCCTTTTTTTTTTTCCAGACCTGTCAAAACTTTACCTTTCCAATTAGGTGATCCAAAAAGACCTACTCCTGACACTCAGAAAGATCATTAGATGTAAAaGACAACCATTCTTGAATAGTTTTTTATTTTTATATAAACCGTTTTCTTCTCACAG
